# JNK Signaling Regulates Cellular Mechanics of Cortical Interneuron Migration

**DOI:** 10.1523/ENEURO.0132-20.2020

**Published:** 2020-08-20

**Authors:** Skye E. Smith, Nicholas K. Coker, Eric S. Tucker

**Affiliations:** 1 Department of Neuroscience, West Virginia University School of Medicine, Morgantown, WV 26506; 2 Biochemistry and Molecular Biology Graduate Program, West Virginia University School of Medicine, Morgantown, WV 26506; 3 WVU Rockefeller Neuroscience Institute, West Virginia University School of Medicine, Morgantown, WV 26506

**Keywords:** cerebral cortex, development, GABAergic interneuron, intracellular signaling, live imaging, neuronal migration

## Abstract

Aberrant migration of inhibitory interneurons can alter the formation of cortical circuitry and lead to severe neurologic disorders including epilepsy, autism, and schizophrenia. However, mechanisms involved in directing the migration of interneurons remain incompletely understood. Using a mouse model, we performed live-cell confocal microscopy to explore the mechanisms by which the c-Jun NH_2_-terminal kinase (JNK) pathway coordinates leading process branching and nucleokinesis, two cell biological processes that are essential for the guided migration of cortical interneurons. Pharmacological inhibition of JNK signaling disrupts the kinetics of leading process branching, rate and amplitude of nucleokinesis, and leads to the rearward mislocalization of the centrosome and primary cilium to the trailing process. Genetic loss of *Jnk* from interneurons also impairs leading process branching and nucleokinesis, suggesting that important mechanics of interneuron migration depend on the intrinsic activity of JNK. These findings highlight key roles for JNK signaling in leading process branching, nucleokinesis, and the trafficking of centrosomes and cilia during interneuron migration, and further implicates JNK signaling as an important mediator of cortical development.

## Visual Abstract

**Figure:**
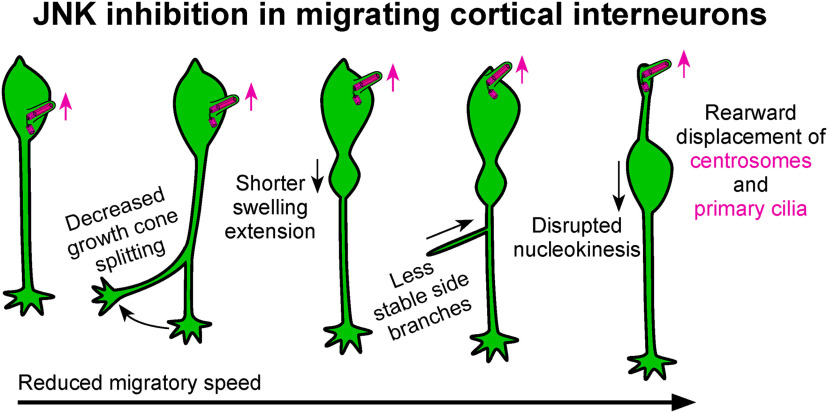

## Significance Statement

Unlike their excitatory counterparts, inhibitory interneurons are generated in the ventral forebrain and migrate long distances to reach the cerebral cortex. Although many factors influencing the guided migration of cortical interneurons have been elucidated, the role of intracellular signaling pathways in interneuron migration have remained elusive. Here, we show with single cell resolution that the c-Jun NH_2_-terminal kinase (JNK) signaling pathway coordinates multiple cellular mechanisms that direct the migration of cortical interneurons, including leading process branching, nucleokinesis, and the subcellular positioning of the centrosome-cilia complex. Furthermore, we show that cortical interneuron migration depends on the intrinsic activity of JNK. These results provide new insight into the cellular and molecular mechanisms controlling cortical interneuron migration in normal and pathologic brain development.

## Introduction

During embryonic development, cortical interneurons are born in the medial ganglionic eminence (MGE) and caudal ganglionic eminence (CGE) of the ventral forebrain and then migrate long distances to reach the place of their terminal differentiation in the overlying cerebral cortex (Wichterle et al., 1999; Nery et al., 2002; Xu et al., 2004; Miyoshi et al., 2010). While navigating their environments, cortical interneurons must integrate extracellular guidance cues with intracellular machinery to reach the cortex, travel in tangentially oriented migratory streams, and disembark from streams at the correct time and place to properly infiltrate the cortical plate. Two cellular mechanisms that enable interneurons to make these complex migratory decisions are leading process branching, where cortical interneurons dynamically remodel their leading processes to sense and respond to extracellular guidance cues, and nucleokinesis, where interneurons propel their cell bodies forward in the selected direction of migration (Polleux et al., 2002; Ang et al., 2003; Nadarajah et al., 2003; Moya and Valdeolmillos, 2004; Bellion et al., 2005). Moreover, proper positioning and signaling from two subcellular organelles, the centrosome and primary cilium, have been implicated in the guided migration of cortical interneurons (Higginbotham et al., 2012; Yanagida et al., 2012; Luccardini et al., 2013, 2015). Failure to coordinate these cellular and subcellular events can alter cortical interneuron migration and impair the development of cortical circuitry, which may underlie severe neurologic disorders such as autism spectrum disorder, schizophrenia, and epilepsy (Kato and Dobyns, 2005; Hildebrandt et al., 2011; Meechan et al., 2012; Volk et al., 2015). While progress has been made on elucidating the complex molecular mechanisms underlying nucleokinesis and leading process branching (Tsai and Gleeson, 2005; Baudoin et al., 2012; Godin et al., 2012; Silva et al., 2018), the intracellular signaling pathways that regulate these cellular mechanisms remain largely unknown.

The c-Jun NH_2_-terminal kinases (JNKs) are evolutionarily conserved members of the mitogen-activated protein kinase (MAPK) super-family (Davis, 2000; Chang and Karin, 2001). The JNK proteins are encoded by three genes, *Jnk1* (*Mapk8*), *Jnk2* (*Mapk9*), and *Jnk3* (*Mapk10*). JNKs phosphorylate numerous substrates in response to extracellular stimuli to mediate physiological processes including cellular proliferation, apoptosis, differentiation, and migration (Davis, 2000).

JNK signaling has been implicated in multiple aspects of brain development, including neuronal migration in the cerebral cortex (Hirai et al., 2006; Wang et al., 2007; Westerlund et al., 2011; Yamasaki et al., 2011; Myers et al., 2014, 2020; Zhang et al., 2016). Indeed, our laboratory previously demonstrated that JNK function is required for the proper timing of interneuron entry into the cerebral cortex (Myers et al., 2014), as well as the tangential progression of interneurons in migratory streams (Myers et al., 2020). However, these previous studies did not assess the influence of JNK on the cellular mechanics of individual cortical interneurons during migration.

In the current study, we extend on those findings by using a combination of pharmacological and genetic manipulations in an MGE explant cortical cell coculture assay to demonstrate that interneurons require JNK-signaling to regulate leading process branching and nucleokinesis. JNK-inhibited MGE interneurons dramatically slow their migration while displaying more variable speeds, and exhibit decreased migratory displacement. Concomitantly, JNK-inhibited interneurons display significant defects in leading process branching with decreased growth cone splitting frequency and interstitial side branch duration, as well as disrupted nucleokinesis and swelling dynamics. Genetic ablation of *Jnk* from MGE interneurons results in leading process branching defects, which were similar to pharmacologic inhibition, and nucleokinesis defects, which were distinct. Nevertheless, migratory deficits resulting from interneuron-specific loss of JNK function indicates that interneurons have a cell-intrinsic requirement for JNK signaling during migration. In addition, we discovered a novel role for JNK signaling in the dynamic localization of the centrosome and primary cilium in migrating interneurons. Surprisingly, the centrosomes and the primary cilia of JNK-inhibited interneurons aberrantly localized to the cell body or trailing process, regardless of whether the leading process contained a swelling. These findings implicate the JNK pathway as a key intracellular mediator of leading process branching, nucleokinesis, and organelle dynamics in migrating MGE interneurons.

## Materials and Methods

### Animals

Animals were housed and cared for by the Office of Laboratory Animal Resources at West Virginia University. Timed-pregnant dams [day of vaginal plug = embryonic day (E)0.5] were euthanized by rapid cervical dislocation at E14.5, and mouse embryos were immediately harvested for tissue culture. CF-1 (Charles River) or C57BL/6J dams (stock #000664; The Jackson Laboratory) were crossed to hemizygous *Dlx5/6-Cre-IRES-EGFP* (*Dlx5/6-CIE*; Stenman et al., 2003) males maintained on a C57BL/6J background to achieve timed pregnancies at E14.5. To generate JNK triple knock-out embryos at E14.5, *Jnk1^fl/fl^*; *Jnk2*^−/−^; *Jnk3*^−/−^ dams were crossed to *Dlx5/6-CIE*; *Jnk1^fl^*^/+^*; Jnk2*^−/−^; *Jnk3*^+/−^ males maintained on a C57BL/6J background. All animal procedures were performed in accordance to protocols approved by the Institutional Animal Care and Use Committee at West Virginia University.

### MGE explant cortical cell coculture

Eight-well chamber coverslip slides (Thermo Fisher Scientific 155411) were coated with a solution of poly-L-lysine (Sigma P5899) and laminin (Sigma L2020) diluted in sterile water (Polleux and Ghosh, 2002), incubated overnight at 37°C with 5% CO_2_, and rinsed with sterile water before cell plating. E14.5 *Dlx5/6-CIE*+ and *Dlx5/6-CIE*- embryos were sorted by GFP fluorescence and dissected in ice-cold complete HBSS (cHBSS; Tucker et al., 2006). Cortices were dissected from the negative brains and pooled together for dissociation (Polleux and Ghosh, 2002). After dissociation, 250 µl of cell suspension diluted to 1680 cells/µl was added to each well and allowed to settle for 2 h. MGE explants were dissected from GFP+ brains and plated on top of cortical cells. Cultures were grown for 24 h before treatments and live imaging. Two E14.5 timed-pregnant dams were used for each genetic experiment. *Dlx5/6-CIE*+ and *Dlx5/6-CIE*- embryos were obtained from a *Dlx5/6-Cre-IRES-EGFP* x C57BL/6J cross, while *cTKO* embryos were obtained by crossing a *Dlx5/6-CIE*; *Jnk1^fl^*^/+^*; Jnk2*^−/−^; *Jnk3*^+/−^ male to a *Jnk1^fl/fl^*; *Jnk2*^−/−^; *Jnk3*^−/−^ dam. MGE explants from *Dlx5/6-CIE*+ wild-type (WT) and *cTKO* embryos were dissected and plated into separate wells containing a monolayer of *Dlx5/6-CIE*- WT cortical cells. Cultures were grown 24 h before live imaging.

### Electroporations

Intact ventral forebrains were microdissected from *Dlx5/6-CIE+* embryos and placed on thin slices of 3% low-melting point agarose (Fisher BP165-25) in cHBSS. Agar slices containing ventral forebrain tissue were placed onto a positive genepaddles electrode (5 × 7 mm; Harvard Apparatus Inc #45-0123) from a BTX ECM 830 squarewave electroporation system under a stereo microscope. Endotoxin-free plasmid DNA (1–3 mg/ml) for Cetn2-mCherry and Arl13b-tdTomato (gift from Eva Anton) was injected into the MGE with a picospritzer (6 ms/spritz; General Valve Picospritzer II), a negative genepaddles electrode (5 × 7 mm; Harvard Apparatus Inc #45-0123) containing a droplet of cHBSS was lowered to the tissue, and electroporated (5 × 60 mV/5-ms pulse length/200-ms interval pulses). Electroporated MGE explants were then dissected, plated as above, and grown for 48 h before imaging.

### Live imaging experiments

Cultures were treated with pre-warmed 37°C serum-free media containing a 1:1000 dilution of DMSO for vehicle control or 20 μm SP600125 pan-JNK inhibitor (Enzo Life Sciences BML-EI305-0010) and immediately transferred to a Zeiss 710 Confocal Microscope with stable environmental controls maintained at 37°C with 5% humidified CO_2_. Multiposition time-lapse z-series were acquired at 10-min intervals over a 12-h period with a 20× Plan-Apo objective (Zeiss) for overall migration analysis, nucleokinesis distance, and swelling distance measurements. For measurements requiring higher temporal and spatial resolution, such as swelling duration, branch dynamics, and visualization of subcellular structures in electroporated cells, cultures were imaged using multiposition time-lapse z-series at 2- to 2.5-min intervals over a 4- to 10-h period with a 40× C-apochromat 1.2-W M27 objective (Zeiss).

### Live imaging analyses

4D live imaging movies were analyzed using Imaris 9.5.1 (Bitplane) software. Movies collected at 20× were evaluated in the first 12 h of each recording. Individual interneurons were manually tracked by placing a “spot” in the center of the cell body in each frame of the movie for a minimum of 4 h. Tracks were discontinued if a cell remained stationary for 60 contiguous minutes, or if the tracked cell could no longer be unambiguously identified. All tracks from each movie were averaged together for dynamic analyses, which included migratory speed, distance, displacement, and track straightness. Displacement was normalized to the minimum track length of 4 h to account for differences in the total times of tracked cells. For pharmacology data, a minimum of 10 cells were tracked from *n* = 11 movies 127 cells/condition) obtained over four experimental days. Genetic data were acquired from a minimum of 10 cells measured from *n* = 13 control movies (five WT embryos, 130 cells) and *n* = 12 conditional triple knock-out (*cTKO*) movies (five *cTKO* embryos, 120 *cTKO* cells), each obtained over four experimental days.

The distance of each nucleokinesis event was measured from the front of the cell body before and after translocation. For swelling analyses, a swelling was defined as a cytoplasmic dilation in the leading process. Swelling distance was measured from the front of the cell body to the most distal portion of the swelling. For pharmacological data, 50 cells were measured from *n* = 10 movies obtained over four experimental days in each condition. For genetic data, nucleokinesis distance measurements included 50 cells sampled from *n* = 10 movies obtained over four experimental days (five *cTKO* embryos, five WT embryos). Pharmacological swelling duration data were obtained from 43 control cells measured from *n* = 10 control movies, and 53 treated cells from *n* = 6 SP600125 movies collected over four experimental days. Genetic swelling duration was obtained from 37 WT cells from *n* = 6 WT movies with five WT embryos, and 38 *cTKO* cells from *n* = 6 *cTKO* movies obtained over four experimental days with four *cTKO* embryos.

For branching analyses, a growth cone split was defined as a bifurcation emerging from the tip of the leading process. An interstitial side branch was defined as a branch arising from the leading process that did not originate from the growth cone. Interneurons had to remain in frame and distinguishable from surrounding cells for a minimum of 3 h to be included in branching analyses. Interstitial side branches had to form *de novo*, persist for a minimum of 10 min, and the branch could not become the new leading process. In pharmacological experiments, frequency data for interstitial side branch formation were obtained by measuring *n* = 19 control cells from eight movies and *n* = 19 SP600125 cells from 10 movies recorded over five experimental days. Data for interstitial side branch duration were obtained from *n* = 52 branches from 14 control cells and *n* = 52 branches from 18 SP600125 cells measured from 10 movies/condition collected over five experimental days. Data for growth cone splitting were obtained from *n* = 19 control cells from eight movies and *n* = 19 SP600125 cells from 10 movies collected over five experimental days. In genetic experiments, data for interstitial side branch frequency were obtained by measuring *n* = 11 cells from six movies/condition collected over four experimental days. Data for side branch duration were obtained from *n* = 34 branches in 10 WT cells and *n* = 28 branches in 10 *cTKO* cells recorded from six movies/condition collected over four experimental days. The frequency of growth cone splitting in genetic experiments were obtained from *n* = 11 cells in six movies/genotype collected over four experimental days.

For electroporation experiments, centrosome and ciliary localization and distance from the front of the cell body were manually tracked and recorded using Imaris software. For centrosome measurements, *n* = 20 cells were analyzed from 11 movies in each condition obtained over five experimental days. For primary cilia measurements, *n* = 20 cells were analyzed from 15 movies in each condition obtained over six experimental days. Two-way ANOVA followed by Fisher’s LSD *post hoc* analyses were performed to determine statistical differences for organelle localization (Prism Version 8 using GraphPad Software). Statistical significances were determined by χ^2^ test for the presence of organelles to a formed swelling over time (Prism Version 8 using GraphPad Software). Two-tailed unpaired Student’s *t* tests were used to determine statistical differences between groups for distance measurements. Length of primary cilia were measured in Imaris based on Arl13b-tdTomato expression. Any cilia that extended outside of the Z-stack were excluded from analyses. Data represent the average ciliary length measured over time per cell. A total of *n* = 17 control and *n* = 15 SP600125 cells collected over 13 movies and six experimental days were analyzed. Statistical differences between ciliary length measurements were determined by two-tailed unpaired Student’s *t* tests. Confocal micrographs were uniformly adjusted for levels, brightness, and contrast in Imaris for movie preparation, and Adobe Photoshop for figure images.

### Statistics

Data were analyzed and graphs were produced using Prism Version 8 (GraphPad Software) or estimation coding software (Ho et al., 2019). For estimation statistics, data are presented as Gardner–Altman estimation plots. A total of 5000 bootstrap samples were taken, and the 95% confidence interval is bias corrected. For graphs not containing estimation statistics, data are presented in scatter plots with solid lines corresponding to the mean ± SEM as indicated in figures and results text. Statistical details can be found in Table 1.

**Table 1:** Summary of statistical analyses

						Estimation statistics	
Figure	Measurement	Datastructure	Type of test	Comparison	Statisticalvalue	Two-sidedpermutation*t* test value	Mean DifferenceWith 95% ConfidenceInterval
Fig. 1*D*	Maximum migration speed	Normal	Unpaired *t* test	Control vs SP	*p* = 1.68E-10; *t*_(20)_ = 11.86	0.0	–54.2 [–63.1, –46.0]
Fig. 1*D*	Mean migration speed	Normal	Unpaired *t* test	Control vs SP	*p* = 1.68E-09; *t*_(20)_ = 10.38	0.0	–28.1 [–33.8, –23.6]
Fig. 1*D*	Minimum migration speed	Normal	Unpaired *t* test	Control vs SP	*p* = 0.0000717; *t*_(20)_ = 4.98	0.0	–4.68 [–6.76, –3.08]
Fig. 1*E*	Speed variation	Normal	Unpaired *t* test	Control vs SP	*p* = 0.000188; *t*_(20)_ = –4.56	0.0002	0.136 [0.0827, 0.196]
Fig. 1*F*	Displacement	Normal	Unpaired *t* test	Control vs SP	*p* = 4.73E-07; *t*_(20)_ = 7.29	0.0	–81.2 [–1.06e+02, –63.6]
Fig. 1*G*	Straightness	Normal	Unpaired *t* test	Control vs SP	*p* = 0.451; *t*_(20)_ = 0.769	0.389	–0.0282 [–0.0955, 0.03]
Fig. 2*C*	Maximum leadingprocess length	Normal	Unpaired *t* test	Control vs SP	*p* = 0.977; *t*_(18)_ = –0.0294	0.975	0.177 [–10.7, 11.7]
Fig. 2*C*	Mean leading processlength	Normal	Unpaired *t* test	Control vs SP	*p* = 0.947; *t*_(18)_ = 0.0668	0.947	–0.257 [–6.85, 7.48]
Fig. 2*C*	Minimum leadingprocess length	Normal	Unpaired *t* test	Control vs SP	*p* = 0.911; *t*_(18)_ = –0.112	0.917	0.413 [–6.12, 7.32]
Fig. 2*D*	Frequency of growthcone splits	Normal	Unpaired *t* test	Control vs SP	*p* = 0.0169; *t*_(34)_ = 2.51	0.0146	–0.681 [–1.13, –0.121]
Fig. 2*G*	Frequency of side branches	Normal	Unpaired *t* test	Control vs SP	*p* = 0.900; *t*_(34)_ = 0.126	0.897	0.0347 [–0.479, 0.499]
Fig. 2*H*	Side branch duration	Normal	Unpaired *t* test	Control vs SP	*p* = 0.016; *t*_(102)_ = 2.46	0.0124	–7.58 [–14.2, –2.12]
Fig. 3*C*	Nucleokinesis distance	Normal	Unpaired *t* test	Control vs SP	*p* = 2.36E-10; *t*_(18)_ = 12.58	0.0	–6.36 [–7.3, –5.42]
Fig. 3*D*	Nucleokinesis frequency	Normal	Unpaired *t* test	Control vs SP	*p* = 1.92E-08; *t*_(18)_ = 8.96	0.0	–0.765 [–0.912, –0.598]
Fig. 3*E*	Pause duration	Normal	Unpaired *t* test	Control vs SP	*p* = 1.45E-07; *t*_(18)_ = –7.89	0.0	9.5 [7.44, 11.8]
Fig. 3*F*	Soma-swelling distance	Normal	Unpaired *t* test	Control vs SP	*p* = 0.001698; *t*_(18)_ = 3.68	0.0022	–1.79 [–2.64, –0.824]
Fig. 3*G*	Swelling duration	Normal	Unpaired *t* test	Control vs SP	*p* = 0.00047; *t*_(14)_ = –4.53	0.0014	7.04 [4.33, 9.76]
Fig. 4*D*	Maximum migration speed	Normal	Unpaired *t* test	WT vs *cTKO*	*p* = 0.981; *t*_(23)_ = 0.0236	0.982	–0.117 [–9.81, 9.2]
Fig. 4*D*	Mean migration speed	Normal	Unpaired *t* test	WT vs *cTKO*	*p* = 0.105; *t*_(23)_ = 1.69	0.11	–5.59 [–12.0, 0.867]
Fig. 4*D*	Minimum migration speed	Normal	Unpaired *t* test	WT vs *cTKO*	*p* = 0.260; *t*_(23)_ = 1.16	0.256	–1.24 [–3.27, 0.771]
Fig. 4*E*	Speed variation	Normal	Unpaired *t* test	WT vs *cTKO*	*p* = 0.022; *t*_(23)_ = –2.46	0.0208	0.057 [0.0166, 0.107]
Fig. 4*F*	Displacement	Normal	Unpaired *t* test	WT vs *cTKO*	*p* = 0.048; *t*_(23)_ = 2.09	0.0492	–29.1 [–54.8, –0.875]
Fig. 4*G*	Straightness	Normal	Unpaired *t* test	WT vs *cTKO*	*p* = 0.027; *t*_(23)_ = 2.37	0.0284	–0.0651 [–0.118, –0.0133]
Fig. 4*H*	Frequency of growthcone splits	Normal	Unpaired *t* test	WT vs *cTKO*	*p* = 0.0454; *t*_(20)_ = 2.13	0.0318	–0.618 [–1.29, –0.183]
Fig. 4*I*	Frequency of sidebranches	Normal	Unpaired *t* test	WT vs *cTKO*	*p* = 0.658; *t*_(20)_ = 0.448	0.654	–0.113 [–0.542, 0.385]
Fig. 4*J*	Side branch duration	Normal	Unpaired *t* test	WT vs *cTKO*	*p* = 0.046; *t*_(60)_ = 2.04	0.044	–8.83 [–17.7, –1.83]
Fig. 5*C*	Nucleokinesis distance	Normal	Unpaired *t* test	WT vs *cTKO*	*p* = 0.028; *t*_(18)_ = 2.39	0.0298	–0.92 [–1.61, –0.17]
Fig. 5*D*	Nucleokinesis frequency	Normal	Unpaired *t* test	WT vs *cTKO*	*p* = 0.00203; *t*_(18)_ = –3.60	0.0012	0.458 [0.262, 0.752]
Fig. 5*E*	Pause duration	Normal	Unpaired *t* test	WT vs *cTKO*	*p* = 0.000464; *t*_(18)_ = 4.27	0.0002	–5.09 [–7.84, –3.27]
Fig. 5*F*	Swelling duration	Normal	Unpaired *t* test	WT vs *cTKO*	*p* = 0.00257; *t*_(10)_ = 3.99	0.0012	–2.61 [–3.94, –1.54]
Fig. 6*D*	Centrosome localization	Normal	Two-way ANOVA(*post hoc* Fisher’sexact test)	Control vs SP	*p* < 0.0001; *F*_(2,114)_ = 13.82	NA	NA
Fig. 6*E*	Centrosome presencein swelling	Normal	χ^2^	Control vs SP	*p* < 0.0001; Χ^2^_(331.1, 1)_ =18.20	NA	NA
Fig. 6*F*	Centrosome maximumdistance forward	Normal	Unpaired *t* test	Control vs SP	*p* = 0.028; *t*_(38)_ = 2.30	0.0256	–3.2 [–5.82, –0.79]
Fig. 6*F*	Centrosome maximumdistance behind	Normal	Unpaired *t* test	Control vs SP	*p* = 0.000015; *t*_(38)_ = 4.97	0	–10.3 [–14.6, –6.49]
Fig. 7*C*	Cilia localization	Normal	Two-way ANOVA(*post hoc* Fisher'sexact test)	Control vs SP	*p* < 0.0001; *F*_(2,114)_ = 12.13	NA	NA
Fig. 7*D*	Cilia presence in swelling	Normal	χ^2^	Control vs SP	*p* < 0.0001; Χ^2^_(314.2,1)_ = 17.72	NA	NA
Fig. 7*E*	Cilia maximum distanceforward	Normal	Unpaired *t* test	Control vs SP	*p* = 0.0094; *t*_(38)_ = 2.76	0.0094	–4.16 [–7.0, –1.61]
Fig. 7*E*	Cilia maximum distance behind	Normal	Unpaired *t* test	Control vs SP	*p* = 0.017; *t*_(38)_ = 2.51	0.0152	–6.38 [–11.6, –1.97]
Fig. 7*F*	Cilia length	Normal	Unpaired *t* test	Control vs SP	*p* = 0.558; *t*_(30)_ = 0.592	0.575	–0.0983 [–0.439, 0.195]
Extended Data Fig. 1-1*A*	Mean migration speed all cells	Normal	Unpaired *t* test	Control vs SP	*p* = 4.65E-53; *t*_(252)_ = 19.74	0.0	–28.4 [–31.1, –25.5]
Extended Data Fig. 1-1*B*	Maximum migration speed	Normal	Unpaired *t* test	Control vs SP	*p* = 0.0053; *t*_(25)_ = 3.05	0.0042	–24.0 [–41.8, –10.6]
Extended Data Fig. 1-1*B*	Mean migration speed	Normal	Unpaired *t* test	Control vs SP	*p* = 0.559; *t*_(25)_ = 0.592	0.545	–0.299 [–1.28, 0.636]
Extended Data Fig. 1-1*B*	Minimum migration speed	Normal	Unpaired *t* test	Control vs SP	*p* = 0.209; *t*_(25)_ = –1.29	0.210	1.39 [–0.708, 3.25]
Extended Data Fig. 1-1*C*	Speed variation	Normal	Unpaired *t* test	Control vs SP	*p* = 0.0038; *t*_(25)_ = 3.19	0.0028	–0.187 [–0.317, –0.0848]
Extended Data Fig. 1-1*D*	Displacement	Normal	Unpaired *t* test	Control vs SP	*p* = 0.158; *t*_(25)_ = –1.45	0.172	15.6 [–5.9, 35.7]
Extended Data Fig. 1-1*E*	Straightness	Normal	Unpaired *t* test	Control vs SP	*p* = 0.146; *t*_(25)_ = –1.49	0.162	0.104 [–0.0304, 0.238]
Extended Data Fig. 1-1*F*	Soma-swelling distance	Normal	Unpaired *t* test	Control vs SP	*p* = 0.0017; *t*_(25)_ = 3.51	0.0008	–2.3 [–3.7, –1.19]
Extended Data Fig. 1-1*G*	Average nucleokinesis distance	Normal	Unpaired *t* test	Control vs SP	*p* = 8.26E-06; *t*_(25)_ = 5.58	0.0	–3.94 [–5.36, –2.6]
Extended Data Fig. 1-1*H*	<10-µm nucleokinesis distance	Normal	Unpaired *t* test	Control vs SP	*p* = 0.239; *t*_(25)_ = –1.20	0.236	0.259 [–0.113, 0.719]
Extended Data Fig. 1-1*I*	>10-µm nucleokinesis distance	Normal	Unpaired *t* test	Control vs SP	*p* = 0.00094; *t*_(25)_ = 3.75	0.0004	–2.73 [–4.26, –1.43]
Extended Data Fig. 1-1*J*	Nucleokinesis frequency	Normal	Unpaired *t* test	Control vs SP	*p* = 0.382; *t*_(25)_ = 0.890	0.381	–0.111 [–0.345, 0.123]
Extended Data Fig. 1-1*K*	Pause duration	Normal	Unpaired *t* test	Control vs SP	*p* = 0.805; *t*_(25)_ = –0.250	0.805	0.363 [–2.62, 2.85]
Extended Data Fig. 1-1*L*	>10-µm nucleokinesis frequency	Normal	Unpaired *t* test	Control vs SP	*p* = 0.087; *t*_(25)_ = 1.78	0.098	–0.233 [–0.458, 0.0489]
Extended Data Fig. 1-1*M*	Frequency of growth cone splits	Normal	Unpaired *t* test	Control vs SP	*p* = 0.00398; *t*_(8)_ = 3.99	0.0096	–1.33 [–1.81, –0.612]
Extended Data Fig. 1-1*N*	Frequency of side branches	Normal	Unpaired *t* test	Control vs SP	*p* = 0.324; *t*_(8)_ = 1.05	0.329	–0.44 [–1.1, 0.369]
Extended Data Fig. 1-1*O*	Side branch duration	Normal	Unpaired *t* test	Control vs SP	*p* = 0.529; *t*_(25)_ = 0.639	0.556	–3.16 [–14.8, 4.59]

All statistical measurements performed in the study, organized by figure panel. The type of measurement, data structure, type of test, group comparison, and statistical values are included for each analysis. Estimation statistics are provided, where applicable. SP = SP600125.

## Results

### Pharmacological inhibition of JNK signaling disrupts MGE interneuron migration *in vitro*

**Figure 1.**
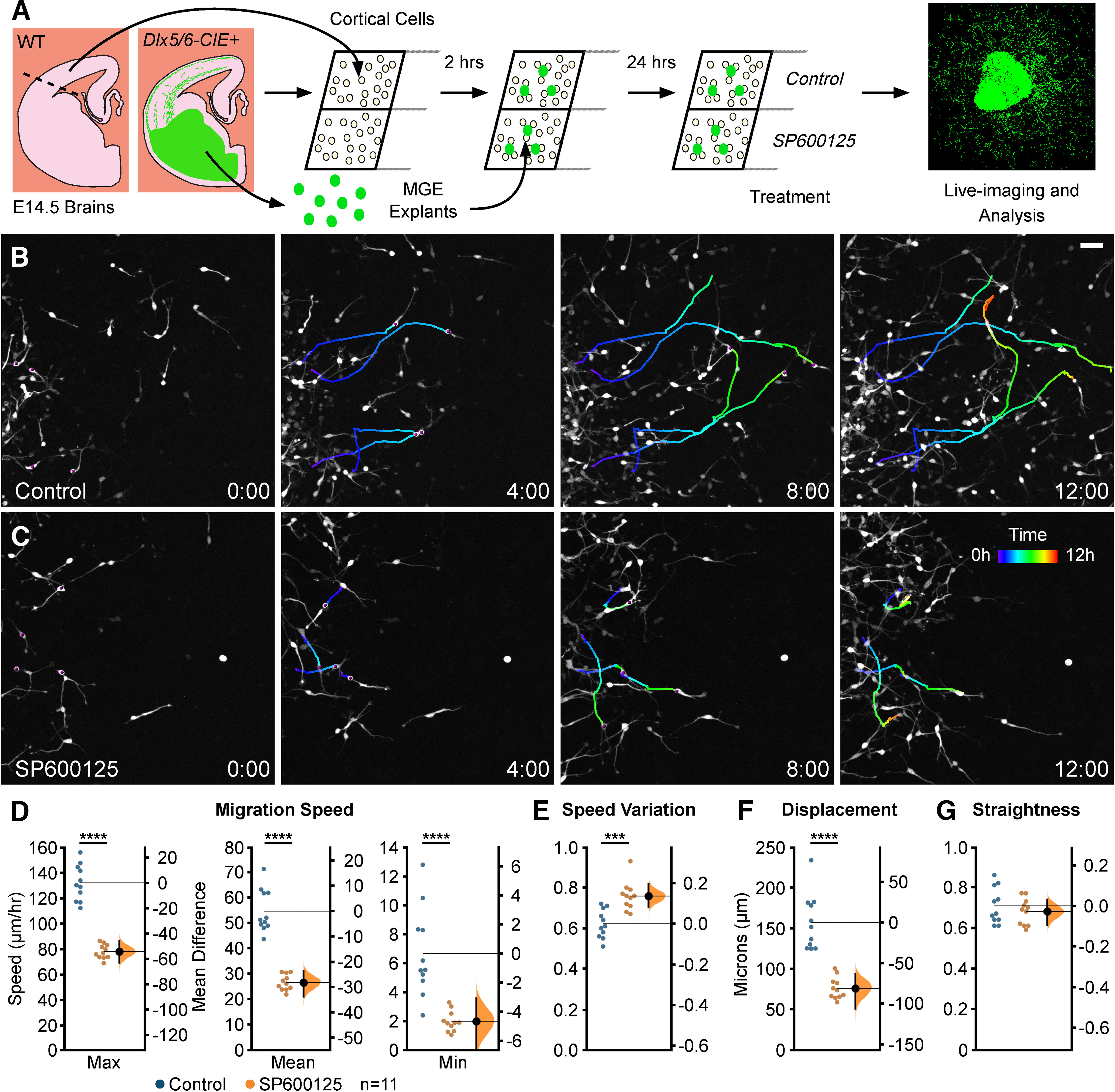
JNK signaling regulates the dynamic migratory properties of MGE interneurons. ***A***, Schematic diagram of MGE explant cortical cell coculture assay with pharmacological inhibition of JNK signaling. ***B***, ***C***, Individual cell tracks (pseudo-colored by time) from four interneurons in control (***B***) or 20 μm SP600125 (***C***) treated cultures imaged live for 12 h. ***D***-***G***, Quantification of interneuron migratory properties revealed significant disruptions in migration speed (***D***), speed variation (***E***), and displacement (***F***), but not straightness (***G***), during JNK inhibition. For each condition, a minimum of 10 cells were tracked from *n* = 11 movies (127 cells/condition) obtained over four experimental days. Data are presented as Gardner–Altman estimation plots. The values of both groups are plotted on the left axes with the mean difference between groups plotted on the right axes as a bootstrap resampling distribution. The mean difference is depicted as a large black dot with the 95% confidence interval indicated by the ends of the vertical error bar; ****p < 0.0001, ***p < 0.001, Student’s *t* test. Time in hours. Scale bar: 50 µm. In addition, a subset of MGE interneurons in control and JNK inhibited conditions were analyzed to determine whether JNK signaling influenced migratory properties of cells traveling at the same average speed (Extended Data Fig. 1-1).

In order to determine the cellular mechanisms by which JNK signaling influences cortical interneuron migration, we examined the migratory dynamics of individual cortical interneurons grown from MGE explants in control or JNK-inhibited conditions. MGE explants from E14.5 *Dlx5/6-Cre-IRES-EGFP* (*Dlx5/6-CIE*)-positive embryos were cultured on top of a *Dlx5/6-CIE*-negative (WT) monolayer of dissociated cortical cells for 24 h (Fig. 1*A*). We then treated cultures with SP600125, a pan inhibitor of JNK signaling (Bennett et al., 2001), or vehicle control and immediately performed live-cell imaging for 12 h (Fig. 1*A*). We treated cultures with 20 μm SP600125 since this concentration of inhibitor caused significant deficiencies in interneuron migration without altering cell viability in previous dosage series and live imaging experiments (Myers et al., 2014, 2020). At the beginning of the imaging period (time 0), the field of view was placed at the distal edge of interneuron outgrowth (Fig. 1*B*,*C*).

10.1523/ENEURO.0132-20.2020.f1-1 Extended Data Figure 1-1 JNK inhibition results in migratory deficits of cortical interneurons regardless of average migratory speed. ***A***, Average migratory speed of individual interneurons in control and JNK inhibited conditions. Red dashed lines highlight cells migrating at the same average speed (35–40 µm/h). Data are individual data points with 5000 bootstrap sampling distribution with the mean difference between control and SP600125-treated conditions on the right *y*-axis. In each condition, *n* = 127 cells from 11 movies obtained over four experimental days. Quantification of migratory properties in interneurons migrating at 35–40 µm/h revealed significant disruptions in maximum migration speed (***B***), and speed variation (***C***), but not displacement (***D***), or straightness (***E***) during JNK inhibition. A total of *n* = 13 control cells and *n* = 14 SP600125-treated cells collected from six to seven movies over four experimental days were within the 35–40 µm/h average speed. ***F–L***, Quantification of nucleokinesis dynamics in control and JNK-inhibition interneurons traveling at the same average migratory speed. Cortical interneurons treated with 20 µm SP600125 have significantly shorter average swelling distances (***F***), smaller average translocation distances (***G***), no change in short translocation distances (***H***), and a significant reduction in large translocation distances (***I***) compared to controls. SP600125 had no effect on average nucleokinesis frequency (***J***), pause duration (***K***), or large translocation distance frequency (***L***) when compared to controls. ***M–O***, Quantification of leading process branching dynamics in control and JNK-inhibited interneurons traveling at the same average migratory speed. Interneurons treated with SP600125 have significantly reduced growth cone split frequencies (***M***), with no disruptions in side branch frequency (***N***) or duration (***O***). In each condition, *n* = 5 cells were analyzed from five movies collected over four experimental days with *n* = 13 control and *n* = 14 SP600125 side branches. Data are presented as Gardner–Altman estimation plots (Student’s *t* test; ****p < 0.0001, ***p < 0.001, **p < 0.01, *p < 0.05). Download Figure 1-1, TIF file.

Many control interneurons migrated into the field of view by 12 h of imaging (Fig. 1*B*; Movie 1), but SP600125-treated cells failed to progress through the frame and appeared to move slower (Fig. 1*C*; Movie 2). To assess potential differences in their migratory dynamics, we tracked individual cells to evaluate how JNK inhibition affects interneuron migration on a single cell level (Fig. 1*B*,*C*, representative cell tracks). The migratory speeds of JNK-inhibited interneurons were significantly slower than controls, including the maximum (values = mean ± SEM; control: 132.28 ± 4.25 µm/h; SP600125: 78.02 ± 1.69 µm/h; *p* = 1.68 × 10^−10^), mean (control: 54.62 ± 2.54 µm/h; SP600125: 26.48 ± 0.94 µm/h; *p* = 1.68 × 10^−9^), and minimum (control: 6.64 ± 0.91 µm/h; SP600125: 1.96 ± 0.21 µm/h; *p* = 7.17 × 10^−5^) migratory speeds (Fig. 1*D*). While JNK-inhibited interneurons migrated slower, speed variation, which is the ratio of track SD to track mean speed, was significantly increased in SP600125-treated conditions (control: 0.62 ± 0.02; SP600125 0.76 ± 0.02; *p* = 0.00,019; Fig. 1*E*). Because of the decrease in migratory speed, the migratory displacement of SP600125-treated interneurons was also significantly reduced compared with control interneurons (control: 156.93 ± 10.37 µm; SP600125: 75.76 ± 4.04 µm; *p* = 4.73 × 10^−7^; Fig. 1*F*). Despite these changes in overall migratory dynamics, JNK-inhibited interneurons displayed no change in their migratory straightness (control: 0.71 ± 0.03; SP600125: 0.68 ± 0.02; *p* = 0.45; Fig. 1*G*). Collectively, these data demonstrate that JNK inhibition impairs the migratory speed and displacement of MGE interneurons in an MGE explant cortical cell coculture assay, confirming previous results in *ex vivo* slice culture experiments (Myers et al., 2020).

Movie 1. Live imaging of MGE interneurons under control conditions. Movie clip 1, E14.5 *Dlx5/6-CIE* MGE explant cortical cell co-culture imaged live for 12 h in control conditions. Interneurons robustly migrate from the margin of the MGE explant to fill the field of view. Movie clip 2, Three MGE interneurons tracked for the duration of the recording. 10.1523/ENEURO.0132-20.2020.video.1

Movie 2. Live imaging of MGE interneurons under 20 μm SP600125 conditions. Movie clip 1, E14.5 *Dlx5/6-CIE* MGE explant cortical cell co-culture imaged for 12 h in SP600125 conditions. Interneurons slowly migrate from the margin of the explant, sparsely populating the field of view. Movie clip 2, Three MGE interneurons tracked for the duration of the recording. 10.1523/ENEURO.0132-20.2020.video.2

### JNK signaling regulates branching dynamics of migrating MGE interneurons

Migrating cortical interneurons repeatedly extend and retract leading process branches to sense extracellular guidance cues and establish a forward direction of movement (Polleux et al., 2002; Bellion et al., 2005; Yanagida et al., 2012). Leading process branching normally occurs through two mechanisms: growth cone splitting at the distal end of the leading process, and formation of interstitial side branches along the length of the leading process (Martini et al., 2009; Lysko et al., 2011).

**Figure 2.**
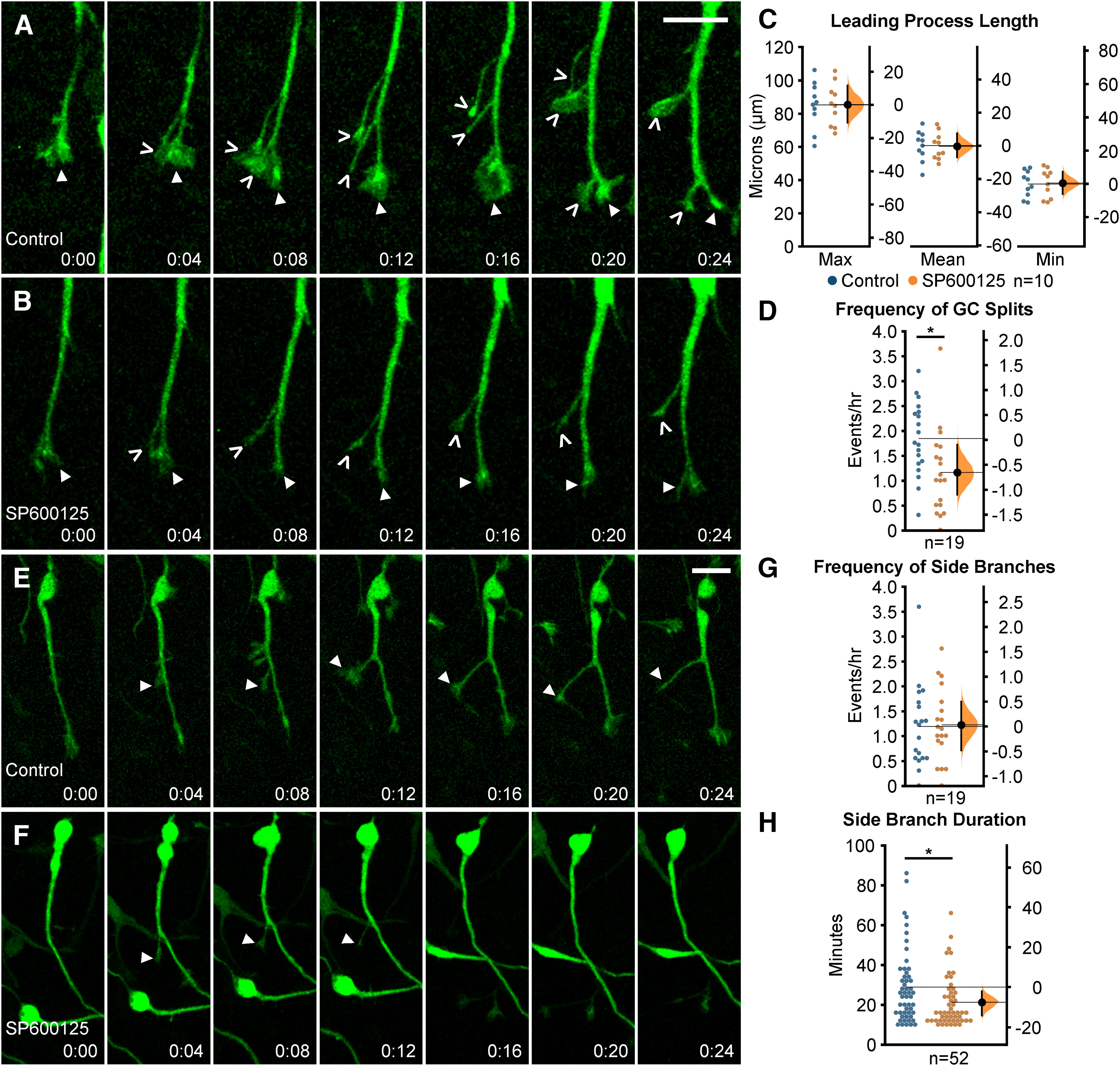
Migrating MGE interneurons require intact JNK signaling for proper leading process branching. ***A***, ***B***, Time series depicting growth cone (GC) splitting from control (***A***) or JNK-inhibited (***B***) MGE interneurons. Closed arrowhead = GC, open arrowhead = new GC branch. ***C***, Quantification of leading process length; *n* = 10 cells were measured from eight movies/condition obtained over four experimental days. ***D***, Quantification of GC splitting frequency; *n* = 19 control cells from eight movies and *n* = 19 SP600125 cells from 10 movies were measured. ***E***, ***F***, Interstitial side branching from control (***E***) or JNK-inhibited (***F***) interneurons. Closed arrowhead = new side branch. ***G***, Quantification of interstitial side branch frequency of control and SP600125-treated interneurons; *n* = 19 control cells from eight movies; *n* = 19 SP600125 cells from 10 movies. ***H***, Quantification of interstitial side branch duration in control and JNK-inhibited conditions; *n* = 52 branches from 14 control cells and 18 SP600125 cells were measured from 10 movies/condition. All branching data were from movies obtained over five experimental days. Data are presented as Gardner–Altman estimation plots; *p < 0.05, Student’s *t* test. Time in minutes. Scale bar: 15 µm.

To determine whether JNK inhibition affected leading process morphology, we first measured the length of leading processes over time from live-imaged *Dlx5/6-CIE* positive MGE interneurons. Maximum (control: 84.96 ± 4.45 µm; SP600125: 85.14 ± 4.02 µm/h; *p* = 0.977), mean (control: 60.39 ± 2.88 µm; SP600125: 60.14 ± 2.56 µm; *p* = 0.947), or minimum lengths (control: 37.40 ± 2.47 µm; SP600125: 37.81 ± 2.72 µm; *p* = 0.912) of leading processes of SP600125-treated interneurons remained unchanged (Fig. 2*C*). However, when we analyzed the dynamic behavior of leading processes, significant differences were found between interneurons in control and SP600125-treated conditions (Fig. 2; Movies 3, 4). In control conditions, migrating MGE interneurons show frequent initiation of new branches from growth cone splitting at the tip of their leading processes (Fig. 2*A*; Movie 3, clip 1). In JNK-inhibited conditions, interneurons still underwent growth cone splitting, but the frequency appeared to be reduced (Fig. 2*B*; Movie 4, clip1). When we measured the rate of growth cone splitting, JNK-inhibited interneurons had a statistically significant reduction compared with controls (control: 1.83 ± 0.17 splits/h; SP600125 1.15 ± 0.20 splits/h; *p* = 0.01; Fig. 2*D*). In addition to branching from their growth cones, MGE interneurons extend and retract interstitial side branches from their leading processes. To determine whether JNK inhibition impacted the frequency and duration of interstitial branching, we measured the rate in which new side branches formed and determined the amount of time each newly generated branch was retained. Both control and SP600125-treated interneurons extended side branches at similar frequencies (control: 1.18 ± 0.19 branches/h; SP600125:1.22 ± 0.17 branches/h; *p* = 0.89; Fig. 2*E–G*; Movies 3, 4, clip 2). However, the duration of time in which *de novo* side branches persisted was significantly reduced in interneurons treated with JNK inhibitor (control: 28.77 ± 2.53 min; SP600125: 21.19 ± 1.76 min; *p* = 0.02; Fig. 2*H*).

Movie 3. Leading process branching dynamics of MGE interneurons under control conditions. Movie clip 1, Interneuron undergoing growth cone splitting over the course of 1 h in control conditions. First growth cone split is marked with an open arrowhead. Movie clip 2, MGE interneuron extending an interstitial side branch (open arrowhead) over the course of 1 h. Only side branches persisting for 10 min were measured (see Materials and Methods). 10.1523/ENEURO.0132-20.2020.video.3

Movie 4. Leading process branching dynamics of MGE interneurons under 20 μm SP600125 conditions. Movie clip 1, SP600125-treated interneuron undergoing one growth cone splitting event (marked by open arrowhead) over the course of 1 h. Movie clip 2, SP600125-treated interneuron extending a short-lived interstitial side branch (open arrowhead) over the course of 1 h. 10.1523/ENEURO.0132-20.2020.video.4

Here, we found that initiation of branching from growth cone splitting was significantly reduced during JNK inhibition. Also, we found that JNK-inhibited interneurons formed side branches at similar rates to controls, but these branches were shorter-lived. Our data indicate that JNK influences branching dynamics of migratory MGE interneurons by regulating the rate of growth cone splitting, and by promoting the stability of newly formed side branches.

### Acute loss of JNK signaling impairs nucleokinesis and cytoplasmic swelling dynamics of migrating MGE interneurons

**Figure 3.**
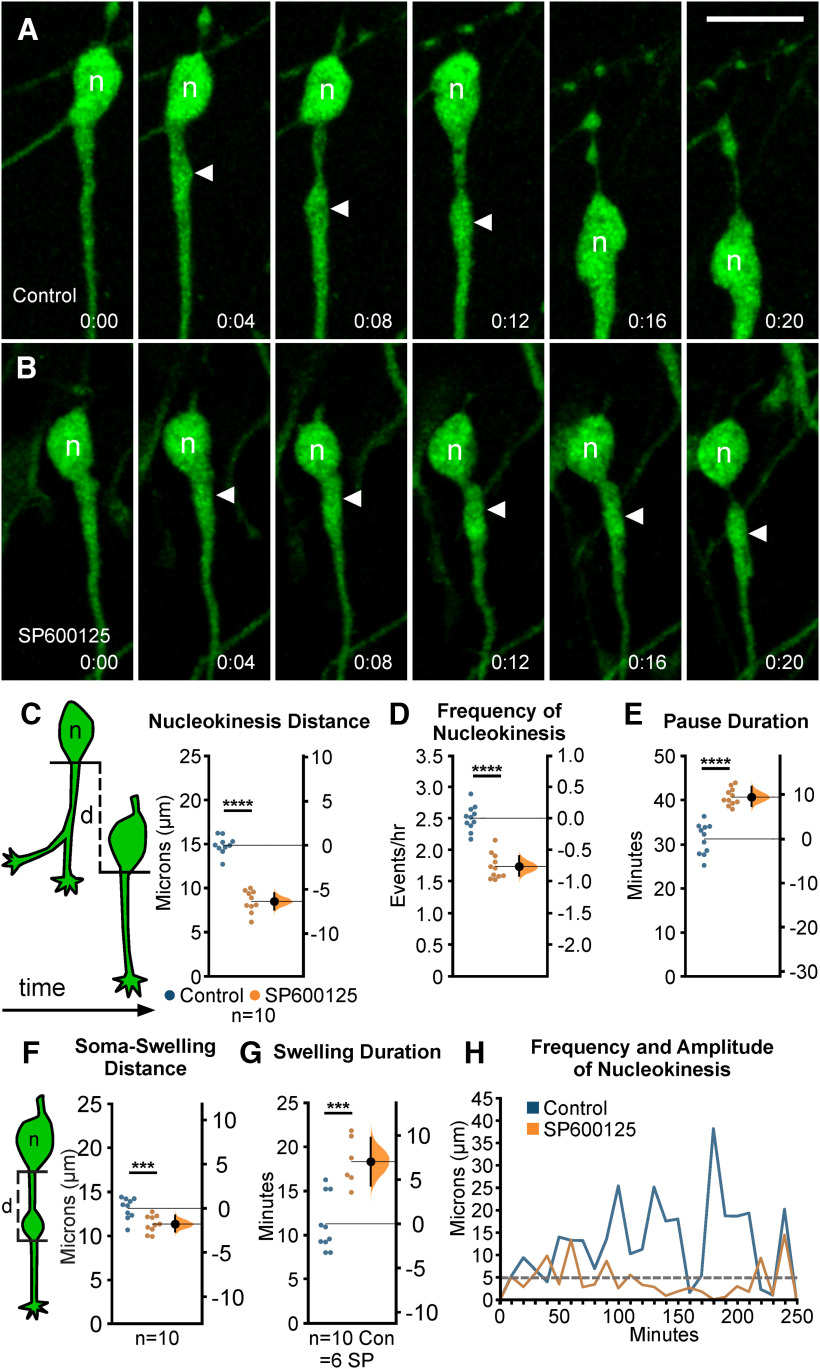
Pharmacological inhibition of JNK signaling impairs nucleokinesis in migrating MGE interneurons. ***A***, ***B***, Time series of a control (***A***) and SP600125-treated (***B***) interneuron undergoing a single cycle of nucleokinesis. Closed arrowhead = leading process swelling, n = nucleus. ***C–E***, Cortical interneurons treated with JNK inhibitor have significantly shorter somal translocation distances (***C***), decreased frequency of nucleokinesis (***D***), and increased pause duration (***E***) compared with controls. ***C***, Cartoon showing how the distance (d) that an interneuron cell body translocates over time was measured. In each condition, 50 cells were measured from *n* = 10 movies obtained over four experimental days. ***F***, Cartoon showing how the distance (d) that a swelling extends from a cell body was measured. JNK-inhibited cells display significantly decreased distance of swelling extension. ***G***, Swelling duration is significantly increased in JNK-inhibited interneurons. A total of 43 control cells were measured from *n* = 10 control movies and 53 treated cells were measured from *n* = 6 SP600125 (SP) movies, each obtained over four experimental days. ***H***, Histogram showing nuclear translocation over time for a single cell in each condition. Distance traveled between two points is plotted and every movement above 5 µm (gray dashed line) is considered to be a nucleokinesis event. Data in ***C–G*** are presented as Gardner–Altman estimation plots; ****p < 0.0001, ***p < 0.001, Student’s *t* test. Time in minutes. Scale bar: 15 µm.

Since pharmacological inhibition of JNK signaling disrupted the overall migratory properties and leading process branching dynamics of MGE interneurons, we further examined the role for JNK in nucleokinesis, an obligate cell biological process in neuronal migration (Bellion et al., 2005; Yanagida et al., 2012). To closely examine the movement of interneuron cell bodies during migration, we imaged cultures at higher spatial and temporal resolution and analyzed the effect of JNK inhibition on nucleokinesis (Fig. 3). Time-lapse recordings show that under control conditions, a single cycle of nucleokinesis starts with the extension of a cytoplasmic swelling into the leading process and ends with the translocation of the cell body into the swelling (Fig. 3*A*; Movie 5, clip 1). Although JNK-inhibited interneurons still engaged in nucleokinesis, the distance of individual nucleokinesis events were disrupted (Fig. 3*B*,*H*; Movie 5, clip 2). When we measured the mean distance that cell bodies translocated over time (Fig. 3*C*, diagram), JNK-inhibited interneurons advanced significantly shorter distances compared with control cells (control: 14.87 ± 0.32 µm; SP600125: 8.50 ± 0.39 µm; *p* = 2.36 × 10^−10^; Fig. 3*C*). Thus, while cell bodies of JNK-inhibited interneurons still translocated forward into the leading process, the distance of their movement was reduced.

Movie 5. Nucleokinesis of MGE interneurons in control and JNK-inhibited conditions. Movie clip 1, Interneuron undergoing multiple nucleokinesis events in control conditions. Movie clip 2, SP600125-treated interneuron engaging in nucleokinesis at a slower rate than control. Open arrowheads mark translocating cell body in both clips. 10.1523/ENEURO.0132-20.2020.video.5

Since cortical interneurons appeared to have slower kinetics of nucleokinesis under JNK inhibition (Fig. 3*B*), we measured the rate of nucleokinesis in control and JNK-inhibited conditions. Upon treatment with SP600125, interneurons completed significantly fewer translocation events per hour (control: 2.50 ± 0.06 events/h; SP600125: 1.73 ± 0.06 events/h; *p* = 1.92 × 10^−8^; Fig. 3*D*). Along with this, interneurons in JNK-inhibited cultures displayed longer pauses between the initiation of nucleokinesis events (control: 31.21 ± 1.05 min; SP600125: 40.71 ± 0.58 min; *p* = 1.45 × 10^−7^; Fig. 3*E*). Because nuclear translocation is preceded by swelling extension, we measured the average distance from the soma to the swelling before translocation (Fig. 3*F*, diagram) and found that SP600125-treated interneurons did not extend cytoplasmic swellings as far as controls (control: 13.13 ± 0.38 µm; SP600125: 11.34 ± 0.30 µm; *p* = 0.002; Fig. 3*F*). Since JNK-inhibited interneurons paused for longer periods of time, we asked whether this was strictly because of delayed nuclear propulsion toward the swelling, or whether the dynamics of swelling extension were also affected. Interneurons treated with SP600125 displayed significantly longer lasting cytoplasmic swellings (control: 11.27 ± 0.99 min; SP600125: 18.31 ± 1.33 min; *p* = 0.0005; Fig. 3*G*), indicating that swelling duration is concomitantly increased with pause duration. Finally, the frequency and amplitude of nuclear translocations that exceed a minimum distance of 5 μm was notably reduced when individual control and JNK-inhibited cells were compared (Fig. 3*H*).

In order to determine whether JNK inhibition altered the migratory properties of interneurons that traveled at similar average speeds, we assessed a subset of control and SP600125-treated MGE interneurons migrating 35–40 µm/h (Extended Data Fig. 1-1*A*). When MGE interneurons traveled at the same average speed, the minimum speed, displacement, and straightness were unaffected by JNK inhibition (Extended Data Fig. 1-1*B–E*). However, the maximum speed that MGE interneurons traveled in SP600125-treated conditions was significantly less than that of controls (Extended Data Fig. 1-1*B*). This decrease in maximum speed under JNK inhibition likely contributed to the significant decrease in the speed variation of MGE interneurons (Extended Data Fig. 1-1*C*). We next asked whether interneurons traveling at the same average speed had impairments to nucleokinesis under JNK inhibition. The average distance that JNK-inhibited MGE interneurons extended a swelling from the cell body as well as the average distance traveled during nucleokinesis were both significantly less than controls (Extended Data Fig. 1-1*F*,*G*), with no changes in frequency or pausing (Extended Data Fig. 1-1*J*,*K*). Moreover, JNK-inhibition led to a significant reduction in the distance of large nucleokinesis events (Extended Data Fig. 1-1*I*). These data likely explain why we found a significant decrease in the maximum speed that MGE interneurons traveled under JNK inhibition. Additionally, when we analyzed leading process branching, MGE interneurons treated with SP600125 had a significant reduction in growth cone split frequency with no changes in side-branch frequency or duration (Extended Data Fig. 1-1*M–O*).

Together, these data point to a role for JNK signaling in regulating the dynamics of nucleokinesis and leading process branching in migrating MGE interneurons, regardless of the average migratory speed that they travel.

### Complete genetic loss of JNK impairs nucleokinesis and leading process branching of migrating MGE interneurons *in vitro*

**Figure 4.**
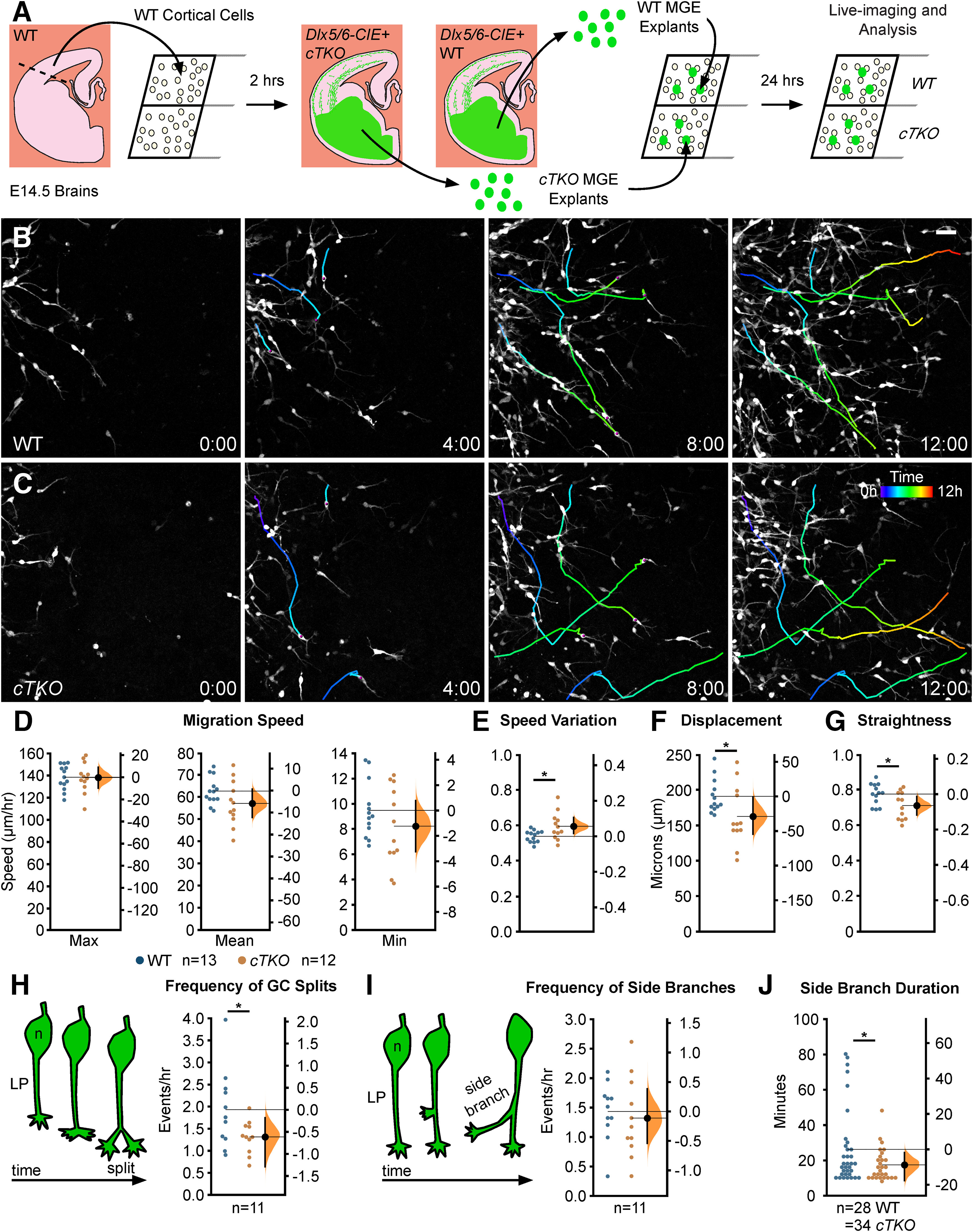
Genetic removal of JNK signaling impairs migratory properties and leading process dynamics of MGE interneurons. ***A***, Diagram of MGE explant assay with *Dlx5/6-CIE+* WT or JNK *cTKO* explants cultured on WT cortical feeder cells. ***B***, ***C***, Four individual cell tracks (pseudo-colored by time) from WT or *cTKO* interneurons imaged live for 12 h. ***D–G***, Quantification of migratory properties reveals no alterations in migratory speed (***D***), but significant disruptions to speed variation (***E***), displacement (***F***), and straightness (***G***) between control and *cTKO* interneurons. A total 120 WT cells were measured from *n* = 13 control movies and 130 *cTKO* cells were measured from *n* = 12 *cTKO* movies, each obtained over four experimental days. ***H***, ***I***, *cTKO* interneurons have significantly decreased growth cone split frequency (***H***) without changes in interstitial side branch frequency (***I***); *n* = 11 cells measured from six movies/condition collected over four experimental days. ***J***, Side branches from *cTKO* interneurons are significantly shorter-lived than controls; *n* = 34 branches were measured from 10 WT cells and *n* = 28 branches were measured from 10 *cTKO* cells recorded from six movies/condition obtained over four experimental days. Data are presented as Gardner–Altman estimation plots; *p < 0.05, Student’s *t* test. Time in hours. Scale bar: 50 µm.

In order to determine that migratory deficits seen with pharmacological inhibition of JNK signaling were specific to the loss of JNK function in cortical interneurons, and not because of JNK inhibition of the cortical feeder cells, we genetically ablated all three JNK genes from interneurons by using mice containing the *Dlx5/6-CIE* transgene to conditionally remove *Jnk1* from *Jnk2;Jnk3* double knock-out embryos (*Dlx5/6-CIE;Jnk1^fl/fl^;Jnk2*^−/−^*;Jnk3*^−/−^). MGE explants from *Dlx5/6-CIE+* WT or conditional triple knockout (*cTKO*) brains were cultured on a WT cortical feeder layer and imaged live (Fig. 4*A*). We tracked individual interneurons over time to assess the overall migratory properties of WT and *cTKO* interneurons (Fig. 4*B*,*C*). While there were no changes in migratory speed (Fig. 4*D*), *cTKO* interneurons exhibited greater variations in migratory speed compared with WT cells (WT: 0.54 ± 0.01; *cTKO*: 0.59 ± 0.02; *p* = 0.02; Fig. 4*E*). We also found that *cTKO* interneurons have shorter migratory displacements than WT interneurons (WT: 195.06 ± 6.80 µm; *cTKO*: 165.99 ± 12.49 µm; *p* = 0.05; Fig. 4*F*). Additionally, the track straightness of *cTKO* interneurons was decreased (WT: 0.77 ± 0.02; *cTKO*: 0.71 ± 0.02; *p* = 0.03; Fig. 4*G*). The combination of increased speed variability and decreased migratory straightness explain why *cTKO* interneurons exhibited shorter migratory displacements. Together, these data indicate that *cTKO* interneurons migrating on WT cortical cells have subtle yet statistically significant deficits in their overall migratory dynamics, which closely match previous findings from *ex vivo cTKO* slice cultures (Myers et al., 2020).

To determine the genetic requirement for JNK signaling in branching, we analyzed leading process branching dynamics of *cTKO* and WT interneurons (Movies 6, 7). *cTKO* interneurons displayed a significant reduction in the frequency of growth cone splitting compared with WT interneurons (WT: 1.92 ± 0.18 splits/h; *cTKO*: 1.30 ± 0.11 splits/h; *p* = 0.04; Fig. 4*H*; Movies 6, 7, clip 1). In addition, genetic removal of JNK signaling from interneurons resulted in no change in side branch initiation (WT: 1.43 ± 0.15; *cTKO*:1.32 ± 0.20 branches/h; *p* = 0.66; Fig. 4*I*), but significant decreases in the duration that side branches persisted (WT: 25.51 ± 3.39 min; *cTKO*:17.29 ± 1.71 min; *p* = 0.05; Fig. 4*J*; Movies 6, 7, clip 2). These data corroborate the findings from our pharmacological analyses and establish a cell intrinsic role for JNK signaling in controlling leading process branching dynamics.

Movie 6. Leading process branching dynamics of WT MGE interneurons. Movie clip 1, WT interneuron undergoing multiple growth cone splitting events (open arrowhead) over the course of 1 h. Movie clip 2, WT interneuron extending an interstitial side branch (open arrowhead) over the course of 1 h. 10.1523/ENEURO.0132-20.2020.video.6

Movie 7. Leading process branching dynamics of *cTKO* MGE interneurons. Movie clip 1, *cTKO* interneuron undergoing fewer growth cone splits (open arrowhead) over the course of 1 h compared to control. Movie clip 2, *cTKO* interneuron extending a short-lived interstitial side branch (open arrowhead) over the course of 1 h. 10.1523/ENEURO.0132-20.2020.video.7

**Figure 5.**
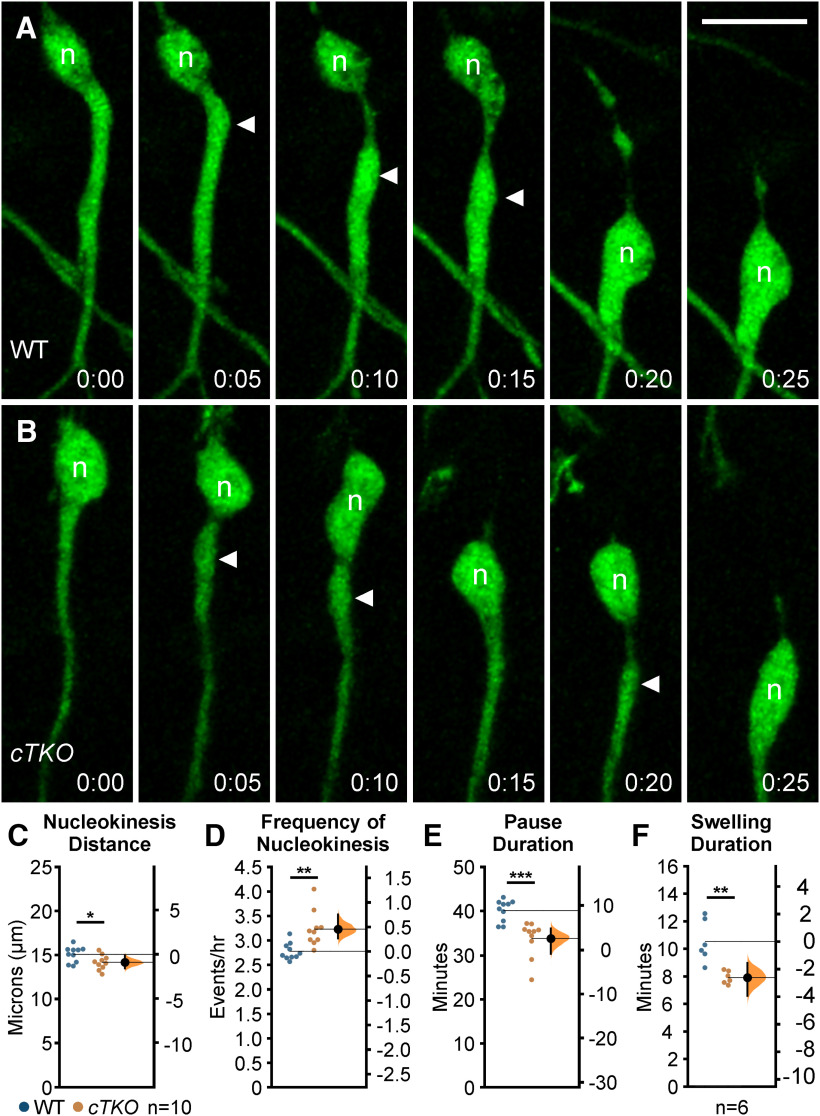
Genetic removal of *Jnk* disrupts nucleokinesis in migrating MGE interneurons. ***A***, WT cortical interneuron undergoing a single nucleokinesis event. ***B***, *cTKO* cortical interneuron completing two nucleokinesis events over the same interval of time. Closed arrowhead = leading process swelling, n = nucleus. ***C–E***, *cTKO* interneurons have significantly decreased translocation distance (***C***), increased translocation frequency (***D***), and decreased pause duration (***E***) compared with WT interneurons. In each condition, 50 cells were measured from *n* = 10 movies obtained over four experimental days. ***F***, *cTKO* interneurons have decreased swelling duration compared with WT interneurons. A total of 37 WT cells were measured from *n* = 6 WT movies and 38 *cTKO* cells were measured from *n* = 6 *cTKO* movies, each obtained over four experimental days. Data are presented as Gardner–Altman estimation plots; ***p < 0.001, **p < 0.01, *p < 0.05, Student’s *t* test. Time in minutes. Scale bar: 15 µm.

Since we found alterations to overall migratory properties and branching dynamics, we next analyzed migrating *cTKO* interneurons for defects in nucleokinesis. Although *cTKO* interneurons engaged in nucleokinesis, the kinetics of nucleokinesis were significantly altered compared with WT interneurons (Fig. 5). The average distance *cTKO* cells traveled forward during nucleokinesis was significantly shorter compared with that of the WT cells (WT: 15.08 ± 0.28 µm; *cTKO*: 14.16 ± 0.26 µm; *p* = 0.03; Fig. 5*A–C*). However, unlike during acute pharmacological inhibition of JNK signaling, *cTKO* interneurons displayed increased rates of nucleokinesis (Fig. 5*A*,*B*; Movie 8). Genetic ablation of JNK signaling in migrating MGE interneurons resulted in increased frequency of translocation events (WT: 2.76 ± 0.05 events/h; *cTKO*: 3.22 ± 0.11 events/h; *p* = 0.002; Fig. 5*D*). While both WT and *cTKO* cells paused after the completion of a nucleokinesis event (after the cell body moves into the swelling), *cTKO* cells spent significantly less time pausing before they extended a new swelling (WT: 32.10 ± 0.62 min; *cTKO* 27.01 ± 1.02 min; *p* = 0.0005; Fig. 5*E*). When we measured the duration of time that cytoplasmic swellings persisted, the swellings in *cTKO* interneurons were significantly shorter-lived (WT: 10.47 ± 0.62 min; *cTKO*: 7.86 ± 0.19 min; *p* = 0.003; Fig. 5*F*). These data likely explain why we did not observe an overall change in migratory speeds between *cTKO* and WT interneurons. While *cTKO* interneurons are not migrating as far during each translocation event they are initiating nucleokinesis at a faster rate, thus moving at similar speeds compared with controls.

Movie 8. Nucleokinesis in WT and *cTKO* MGE interneurons. Movie clip 1, WT interneuron undergoing nucleokinesis. Movie clip 2, *cTKO* interneuron engaging in nucleokinesis at a higher rate than control. Open arrowheads mark translocating cell body in both clips. 10.1523/ENEURO.0132-20.2020.video.8

Collectively, our data indicate that genetic removal of *Jnk* alters the migratory behavior of MGE interneurons, reinforcing our pharmacological findings. While the phenotypes observed with conditional removal of *Jnk* from migrating interneurons were not identical to pharmacological inhibition of JNK signaling, the results from our genetic experiments clearly indicate that interneurons have a cell intrinsic requirement for *Jnk* in leading process branching dynamics and nucleokinesis.

### Subcellular localization and dynamic behavior of the centrosome and primary cilia in migrating MGE interneurons depend on intact JNK-signaling

The cytoplasmic swelling emerges from the cell body during nucleokinesis and contains multiple subcellular organelles involved in the forward movement of cortical interneurons (Bellion et al., 2005; Martini and Valdeolmillos, 2010; Yanagida et al., 2012). One organelle involved in nucleokinesis is the centrosome, which translocates from the cell body into the swelling during nucleokinesis. The centrosome is tethered to the nucleus through a perinuclear cage of microtubules and acts to generate a forward pulling force on the nucleus during nucleokinesis (Bellion et al., 2005; Umeshima et al., 2007). Disruptions in centrosome motility and positioning are thought to underly nucleokinesis defects seen in other studies of neuronal migration (Solecki et al., 2009; Luccardini et al., 2013, 2015; Silva et al., 2018). Since we found significant defects in nucleokinesis of MGE interneurons with both pharmacological and genetic loss of JNK function, we sought to determine whether centrosome dynamics were also disrupted during JNK inhibition.

**Figure 6.**
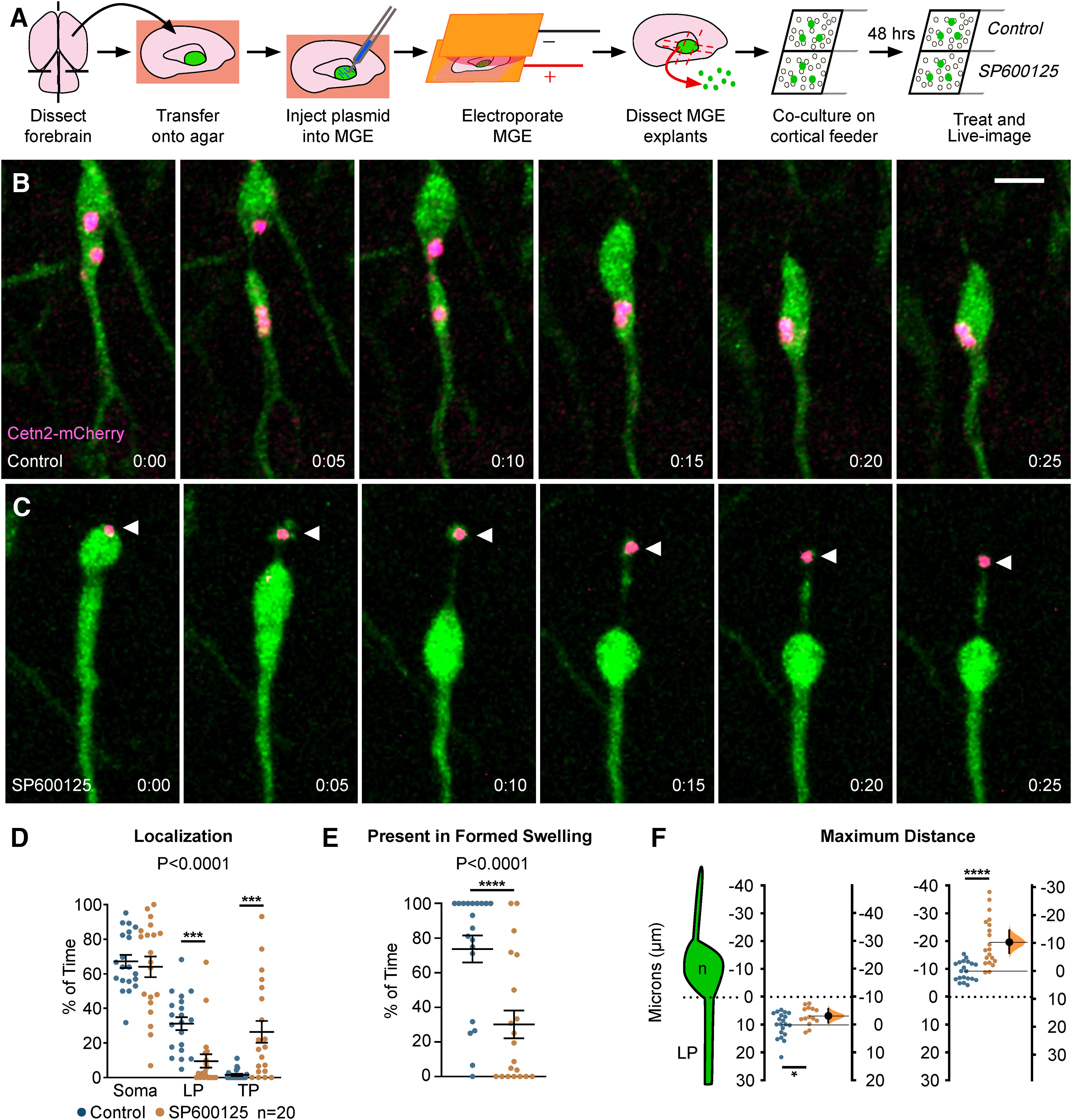
Centrosomes are mislocalized in MGE interneurons during JNK inhibition. ***A***, Diagram depicting *ex vivo* electroporation of MGE tissue and subsequent culture of MGE explants on cortical feeder cells. ***B***, An interneuron expressing a fluorescently tagged centrosome protein (Centrin2; Cetn2-mCherry) shows translocation of the centrosome into the cytoplasmic swelling before nucleokinesis in control conditions. ***C***, A Cetn2-mCherry expressing interneuron treated with SP600125 shows aberrant rearward movement of the centrosome into the trailing process. Arrowhead = Cetn2-mCherry. ***D***, Scatter plot of centrosome distribution over time (centrosome: two-way ANOVA: *F*_(2,114)_ = 13.82; *p* < 0.0001; *p* < 0.0001). Error bars represent mean ± SEM, *post hoc* by Fisher’s LSD ***p < 0.001, **p < 0.01, *p < 0.05. ***E***, Scatter plot depicting centrosome occupancy of a formed swelling over time (χ^2^ test; ****p < 0.0001). Error bars represent mean ± SEM. ***F***, Average maximum distance the centrosome traveled from the soma front (Student’s *t* test; ****p < 0.0001, ***p < 0.001, **p < 0.01, *p < 0.05). In each condition, *n* = 20 cells were measured from 11 movies obtained over five experimental days. Data in ***F*** are presented as Gardner–Altman estimation plots. Time in minutes. Scale bar: 7.5 µm.

To visualize the centrosome and study the role of JNK signaling in centrosome dynamics in migrating MGE interneurons, we live-imaged *Dlx5/6-CIE+* cells expressing a red-fluorescent centrosome marker, Cetn2-mCherry (Fig. 6*A*). In control cells, the centrosome moved correctly into the cytoplasmic swelling (Fig. 6*B*; Movie 9, clip 1), with centrioles occasionally splitting between the soma and swelling preceding nucleokinesis (Fig. 6*B*, frames 0:00–0:10 min), as reported elsewhere (Bellion et al., 2005; Umeshima et al., 2007). Upon JNK-inhibition, the centrosome often maintained a position near the soma regardless of the presence of a swelling (Fig. 6*C*; Movie 9, clips 2, 3). Moreover, in many JNK-inhibited cells, the centrosome moved backwards into the trailing process, even when the cell body translocated forward (Fig. 6*C*; Movie 9, clips 2, 3). When we tracked the positioning of the centrosome over time, the centrosome of JNK-inhibited cells spent significantly more time in the trailing process and less time in the leading process (*p* = 0.0001; Fig. 6*D*). Additionally, when a swelling was formed in front of the soma, the centrosome of JNK-inhibited cells spent significantly less time inside of the swelling than controls (control: 66.64 ± 5.99%; SP600125: 16.08 ± 5.52% of time; *p* = 0.0001; Fig. 6*E*). When we measured the average maximal distance that the centrosome was displaced from the somal front, the centrosome of JNK-inhibited interneurons maintained a significantly closer position to the leading pole of the soma compared with controls (control: 9.93 ± 0.99 µm; SP600125: 6.73 ± 0.88 µm; *p* = 0.03; Fig. 6*F*). This was not surprising since the soma-to-swelling distance in JNK-inhibited interneurons was decreased (Fig. 3*E*). However, when we compared the average maximal rearward distance between the centrosome and somal front, the centrosome of JNK-inhibited interneurons was significantly further behind that of controls (control: 9.40 ± 0.77 µm; SP600125: 19.75 ± 1.94 µm; *p* = 1.48 × 10^−5^; Fig. 6*F*).

Movie 9. Centrosome dynamics in MGE interneurons under control and SP600125-treated conditions. Movie clip 1, Control interneuron electroporated with centrosomal marker Cetn2- mCherry. The centrosome moves from the cell body into the cytoplasmic swelling under control conditions. Movie clip 2, SP600125-treated interneuron electroporated with Cetn2-mCherry. The centrosome moves from the cell body into the trailing process under SP600125 conditions. Movie clip 3, A second SP600125-treated interneuron electroporated with Cetn2-mCherry. The centrosome separates into two centrioles and moves from the cell body into the trailing process and back under SP600125 conditions. Open arrowheads mark Cetn2-mCherry expressing interneurons in all clips. 10.1523/ENEURO.0132-20.2020.video.9

Since we found defects in centrosome dynamics, we wanted to determine whether primary cilia, which normally extend from the mother centriole and house receptors important for the guided migration of cortical interneurons (Baudoin et al., 2012; Higginbotham et al., 2012), were also perturbed in interneurons following JNK-inhibition. In order to study the localization of cilia in migrating interneurons, we performed live-cell confocal imaging on *Dlx5/6-CIE*+ MGE cells expressing Arl13b-tdTomato, a red-fluorescent cilia marker.

**Figure 7.**
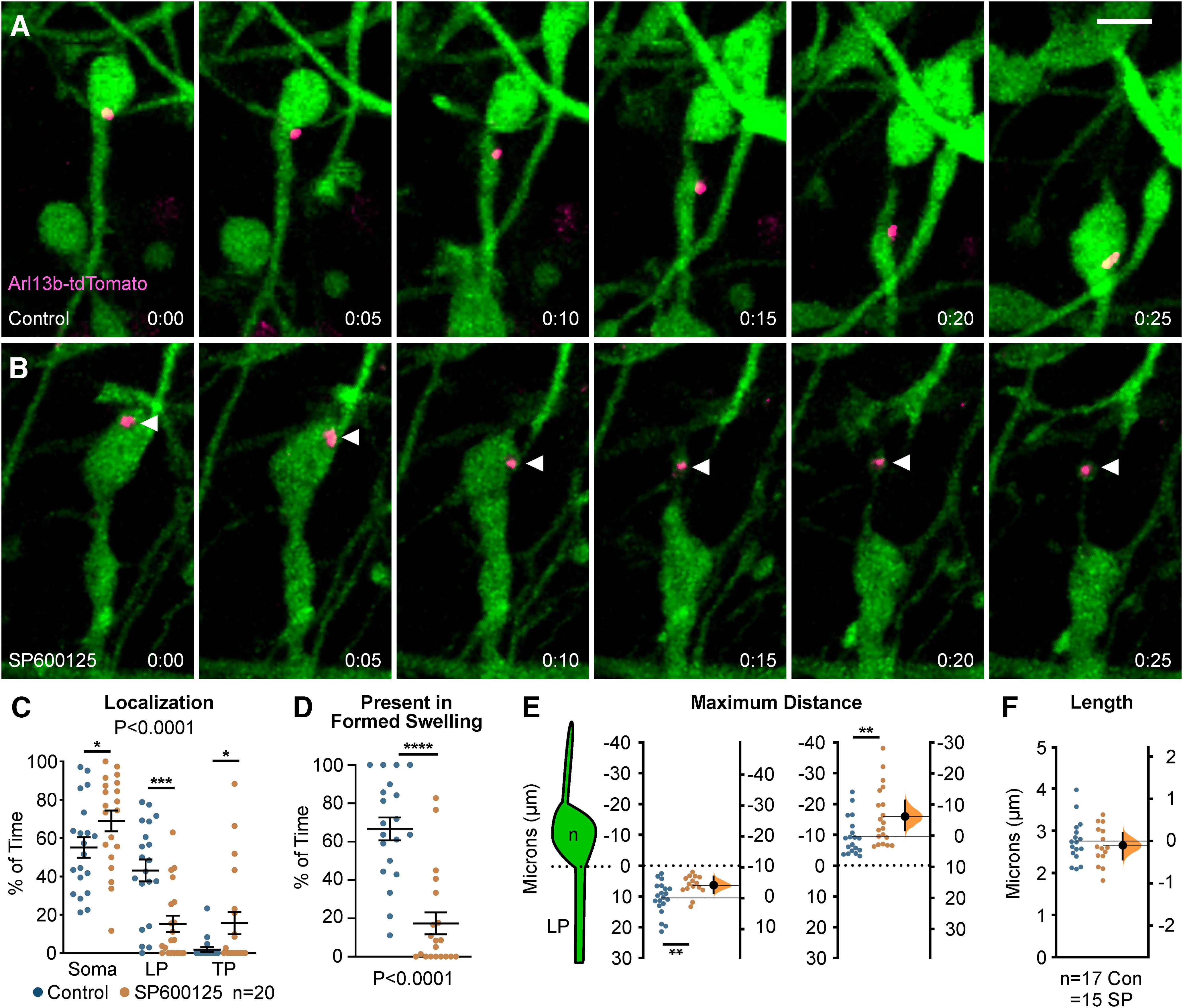
Primary cilium localization in MGE interneurons is disrupted during JNK inhibition. ***A***, An interneuron expressing a fluorescently tagged primary ciliary marker (Arl13b-tdTomato) shows translocation of the primary cilium into the cytoplasmic swelling before nucleokinesis in control conditions. ***B***, An interneuron expressing Arl13b-tdTomato shows aberrant rearward movement of the primary cilium into the trailing process when treated with SP600125. Arrowhead = Arl13b-tdTomato. ***C***, Scatter plot of primary cilium distribution over time (two-way ANOVA: *F*_(2,114)_ = 12.13; *p* < 0.0001). Error bars represent mean ± SEM, *post hoc* by Fisher’s LSD ***p < 0.001, **p < 0.01, *p < 0.05. ***D***, Scatter plot depicting primary cilium occupancy of a formed swelling over time (χ^2^ test; ****p < 0.0001). ***E***, Average maximum distance the primary cilium traveled from the soma front (Student’s *t* test; **p < 0.01). In each condition, *n* = 20 cells were measured from 15 movies obtained over six experimental days. ***F***, Average ciliary length measured over time. In each condition, *n* = 17 control and *n* = 15 SP600125 (SP) cells were measured over 13 movies and six experimental days. Data in ***E***, ***F*** are presented as Gardner–Altman estimation plots. Student’s *t* test; ****p < 0.0001, ***p < 0.001, **p < 0.01, *p < 0.05. Time in minutes. Scale bar: 7.5 µm.

Almost identical to that of our centrosome analyses, we found significant alterations in the dynamic positioning of primary cilia in migrating MGE interneurons (Fig. 7). In control cells, the primary cilium moved into the cytoplasmic swelling before nuclear translocation (Fig. 7*A*; Movie 10, clip 1). However, on JNK inhibition, the cilium was frequently positioned in the soma and often moved into the trailing process as the cell body translocated forward (Fig. 7*B*; Movie 10, clips 2, 3). Overall, the cilium spent significantly more time in the cell soma and behind the cell in the trailing process, and significantly less time in the leading process of JNK-inhibited cells (*p* = 0.0001; Fig. 7*C*). Additionally, the primary cilia in JNK-inhibited interneurons failed to spend as much time in formed cytoplasmic swellings as controls (control: 73.73 ± 7.81% of time; SP600125: 33.85 ± 8.20% of time; *p* = 0.0001; Fig. 7*D*). When we measured the maximal distance behind the somal front, the cilia of JNK-inhibited interneurons were also positioned further behind the cell body than controls, matching our centrosome findings (control: 9.71 ± 1.16 µm; SP600125: 16.09 ± 2.10 µm; *p* = 0.02; Fig. 7*E*). Despite being mispositioned during JNK inhibition, the length of Arl13b-tdTomato labeled primary cilia remained unchanged over time (control: 2.75 ± 0.12 µm; SP600125: 2.65 ± 0.12 µm; *p* = 0.56; Fig. 7*F*), suggesting that the formation and maintenance of primary cilia were not interrupted. Taken together, our data indicate that JNK signaling plays a critical role in the dynamic movement and positioning of the centrosome and primary cilium in migrating interneurons.

Movie 10. Dynamics of primary cilia in MGE interneurons under control and SP600125-treated conditions. Movie clip 1, Interneuron electroporated with primary ciliary marker Arl13b-tdTomato. The cilium moves from the cell body into the cytoplasmic swelling under control conditions. Movie clip 2, Interneuron electroporated with Arl13b-tdTomato in SP600125-treated conditions. The cilium moves from the cell body into the trailing process under SP600125 conditions. Movie clip 3, A second interneuron expressing Arl13b-tdTomato under SP600125-treated conditions. The primary cilium moves from the cell body into the trailing process and returns to the cell body without ever entering the swelling. Open arrowheads mark Arl13b-tdTomato expressing interneurons in all clips. 10.1523/ENEURO.0132-20.2020.video.10

## Discussion

In the present study, we demonstrated that migrating MGE interneurons rely on the JNK signaling pathway to properly undergo leading process branching and nucleokinesis. Pharmacological inhibition of JNK signaling in an *in vitro* assay resulted in reduced migratory speed and displacement with an increase in speed variation of migrating interneurons. Concomitant with these alterations in migratory properties, JNK-inhibited interneurons displayed decreased initiation of branches arising from growth cone tips, decreased persistence of interstitial side branches, as well as shorter, less frequent nucleokinesis events. Even when comparing interneurons that traveled at the same average speed, we found pronounced decreases in large nucleokinesis events, maximum migratory speeds, and growth cone branching of JNK-inhibited interneurons. Importantly, we extended our pharmacological findings by using a *cTKO* mouse line to completely remove *Jnk* from MGE interneurons and not the cortical feeder cells on which they were grown. *cTKO* interneurons migrating on WT feeder cells had decreased migratory displacement without reductions in overall migratory speed, apparently resulting from migratory trajectories that had more variable speeds and reduced track straightness compared with controls. *cTKO* interneurons displayed decreased growth cone branching and interstitial side branch duration, as well as shorter, more frequent nucleokinesis events. Therefore, our results indicate that MGE interneurons have a cell-intrinsic requirement for JNK in the coordination of leading process branching and nucleokinesis. Finally, we found a novel role of JNK signaling in regulating the dynamic positioning of two organelles involved in nucleokinesis: the centrosome and primary cilium. Centrosomes and primary cilia failed to properly translocate into a leading process swelling and spent significantly more time mislocalized to the trailing process of JNK-inhibited interneurons. In summary, we find that loss of JNK signaling impairs cellular and subcellular kinetics of MGE interneuron migration.

### Cytoskeletal regulation during leading process branching and nucleokinesis of migrating interneurons

Leading process branching and nucleokinesis, the two main features of guided interneuron migration, rely on the coordination of actomyosin and microtubule-based cytoskeletal networks. Leading process branches initially form through membrane protrusions containing a F-actin meshwork, which are then stabilized by microtubules to allow for the emergence of the nascent branch (Martini et al., 2009; Spillane et al., 2011; Lysko et al., 2014; Peyre et al., 2015). Nucleokinesis is thought to be mediated through the combination of the forward pulling forces from microtubules at the front of the cell and pushing forces from actomyosin contraction at the rear (Bellion et al., 2005; Martini et al., 2009; Martini and Valdeolmillos, 2010). While mechanisms underlying these processes are still under investigation, molecular mediators of microtubule and actin dynamics in migrating interneurons have begun to emerge. Interestingly, disruptions to these cytoskeletal regulators result in migratory deficits that are reminiscent of those seen following the loss of JNK function.

For instance, p27^kip1^, a microtubule associated protein, coordinates both actomyosin contraction and microtubule organization to control leading process branching and nucleokinesis in migrating interneurons (Godin et al., 2012). Conditional deletion of *p27^kip1^* from post-mitotic interneurons resulted in slower migratory speed, increased frequency of nucleokinesis, and shorter distance of translocations. Similarly, *cTKO* interneurons had shorter translocation distances and increased rates of nucleokinesis. In addition, p27^kip1^ knock-out interneurons displayed shorter-lived side branches, similar to our findings with both pharmacological and genetic loss of JNK. JNK signaling was reported to regulate p27^kip1^ phosphorylation during cancer cell migration (Kim et al., 2012), suggesting a possible link between JNK signaling and this molecular mediator of cellular migration.

Another important regulator of nucleokinesis and leading process branching is the microtubule associated protein doublecortin (Dcx; Kappeler et al., 2006; Friocourt et al., 2007), which is a downstream target of JNK signaling in neurons (Gdalyahu et al., 2004; Jin et al., 2010). Cortical interneurons lacking Dcx show a decreased duration of interstitial side branches, and significantly shorter nuclear translocation distances with no overall changes in migratory speed (Kappeler et al., 2006), similar to what we found in *cTKO* interneurons. Thus, it is possible that JNK signaling fine-tunes leading process branching and nucleokinesis in cortical interneurons by phosphorylating Dcx.

Recently, the role of the elongator complex, specifically the enzymatic core Elp3, was found to control both leading process branching and nucleokinesis through the regulation of actomyosin activity (Tielens et al., 2016). MGE interneurons devoid of Elp3 displayed nucleokinesis and leading process branching defects strikingly similar to our pharmacological results, including decreased migratory speed, translocation frequency, nucleokinesis amplitude, and frequency of growth cone splitting (Tielens et al., 2016). Moreover, the Elongator complex was found to potentiate JNK activity during cellular stress in HeLa and HEK293 cells (Holmberg et al., 2002; Kojic and Wainwright, 2016). This suggests that the Elongator complex may potentiate the activity of JNK to phosphorylate effector proteins required for proper migration of interneurons.

Despite the possible connections, it is currently unknown whether JNK signaling is linked to the function of cytoskeletal regulators in migrating cortical interneurons. Future studies will be required to unravel the exact molecular mechanisms underlying how JNK coordinates leading process branching and nucleokinesis during cortical interneuron migration.

### Position and function of the centrosome and primary cilium during cortical interneuron migration

During the migration cycle of cortical interneurons, a cytoplasmic swelling containing two interconnected organelles, the centrosome and primary cilium, extends ahead of the soma into the leading process (Bellion et al., 2005; Tsai and Gleeson, 2005). Disruptions to the movement, positioning, or function of these organelles have been found in neurons with migratory deficits (Baudoin et al., 2012; Higginbotham et al., 2012; Luccardini et al., 2013; Guo et al., 2015; Nakamuta et al., 2017; Matsumoto et al., 2019), suggesting centrosomes and cilia play essential roles in neuronal migration.

Migratory olfactory bulb interneurons require DOCK7, a member of the DOCK180 family of atypical Rac/Cdc42 guanine nucleotide exchange factors, for migration (Nakamuta et al., 2017). Knock-down of DOCK7 led to unstable movement of the centrosome from the swelling back into the cell body (Nakamuta et al., 2017), which was attributed to slower migration of olfactory bulb interneurons devoid of DOCK7. We observed similar migratory deficits and disrupted centrosome positioning in MGE interneurons treated with JNK inhibitor. Interestingly, knock-down of DOCK7 was previously shown to reduce JNK phosphorylation during Schwann cell development and migration (Yamauchi et al., 2008, 2011).

Furthermore, inactivation of the cell adhesion molecule N-cadherin from MGE interneurons leads to mislocalization of the centrosome to the rear of the cell body (Luccardini et al., 2013). JNK-inhibition not only impeded the forward progression of centrosomes into the swelling, but also led to their unobstructed movement into the trailing process. Interestingly, JNK-inhibition has been reported to decrease N-cadherin levels and cellular migration of myofibroblasts (De Wever et al., 2004), which suggests a potential role for JNK signaling in the regulation of N-cadherin during migration. While mechanisms that control the positioning of the centrosome in migrating neurons remain to be explored, JNK signaling may help synchronize the activity of cell adhesion molecules, cytoskeletal proteins, and cytoplasmic machinery that are critically involved in centrosome motility.

Finally, disruptions to ciliary proteins including Arl13b, Kif3a, and IFT88 or to the sonic hedgehog (Shh) signal transduction pathway all result in cortical interneuron migratory deficits (Baudoin et al., 2012; Higginbotham et al., 2012; Guo et al., 2017). Conditional deletion of *Arl13b* disrupts the formation of the primary cilium from the centrosome and the localization/transport of key receptors known to be critical for interneuron migration, including C-X-C motif chemokine receptor 4 (Cxcr4), neuregulin-1 receptor (ErbB4), and the serotonin receptor 6 (5-Htr6; Riccio et al., 2009; Wang et al., 2011; Higginbotham et al., 2012). Dominant negative knock-down of Kif3a, a molecular motor required for cilium-specific Shh signal transduction, results in rearward movement of the centrosome of migrating olfactory bulb interneurons (Matsumoto et al., 2019), suggesting that functional primary cilia are necessary for the proper localization of the centrosome-cilium complex. Interestingly, the protein kinase A (PKA) signaling pathway appears to the regulate nucleokinesis through the centrosome-cilia complex of migratory olfactory bulb interneurons, since removal of cyclic AMP production from the primary cilium or delocalization of PKA from the centrosome resulted in disrupted nucleokinesis and centrosome movement (Stoufflet et al., 2019). Thus, cortical interneurons may require the function of signal transduction machinery inside the primary cilium for the centrosome-cilium complex to localize correctly, and to sense and respond to environmental guidance cues that promote directed migration of interneurons. Additionally, cortical interneurons lacking Arl13b exhibited leading process branching defects, suggesting that the primary cilium may have cytoskeletal functions along with its role in transducing guidance signals (Higginbotham et al., 2012). While JNK signaling does not appear to be necessary for the formation or stabilization of the primary cilium, it does control the dynamic subcellular position of the centrosome-ciliary complex during MGE interneuron migration. Future studies are needed to determine whether loss of JNK signaling impairs the localization of centrosome and cilia by disrupting ciliary signaling, and whether mislocalized cilia can compromise the guided migration of cortical interneurons *in vivo*.

### Cellular influences of JNK signaling during cortical interneuron migration

In previously published work from *ex vivo* slice cultures, pharmacological inhibition of JNK signaling led to slowed migration and shorter migratory displacement, while genetic loss of *Jnk* from interneurons did not change migratory speed, but led to shorter displacement distances (Myers et al., 2020). Here, we confirmed the requirement for JNK in interneuron migration using an *in vitro* coculture assay and extended these observations to include a detailed assessment of the individual cellular and subcellular mechanics of leading process branching and nucleokinesis. Loss of JNK function disrupted leading process branching and nucleokinesis of MGE interneurons and led to significant alterations of their migratory properties. The requirement of JNK in interneuron migration could be multifactorial, however, and regulate interneuron migration through intrinsic mechanisms, extrinsic mechanisms, or both. Since SP600125 treatment inhibits JNK function in all cells of the MGE explant cortical cell coculture assay, we cannot exclude the possibility that JNK inhibition disrupts cell-cell interactions between interneurons and the cortical feeder cells on which they are grown. To determine whether migrating MGE interneurons have a cell-autonomous requirement for JNK signaling, we genetically removed *Jnk* from interneurons and cultured them on WT cortical cells. Although we found migratory deficits in *cTKO* interneurons that were indicative of an intrinsic function for JNK, the deficits we uncovered were distinct from pharmacological experiments, suggesting that there may be additive effects when JNK is simultaneously removed from both populations of cells. Both pharmacological inhibition and genetic removal of *Jnk* resulted in consistent leading process branching phenotypes with decreased growth cone splitting and short-lived interstitial side branches. When we analyzed nucleokinesis, however, the kinetics of movement were opposite: JNK-inhibited cells completed nucleokinesis at slower rates, whereas *cTKO* cells completed at faster rates. These data imply that cortical interneuron migration is dependent on both intrinsic and extrinsic requirements for JNK signaling. Indeed, regulation of leading process branching in cortical interneurons may be dominated by an intrinsic function of JNK signaling, since the exclusive genetic loss of JNK from interneurons matches the combined pharmacological loss of JNK from both interneurons and cortical cells. Meanwhile, JNK signaling may influence nucleokinesis through both cell-intrinsic and extrinsic mechanisms, which could explain departures in the rates of nucleokinesis between JNK-inhibited and *cTKO* interneurons. While the exact mechanisms that cortical interneurons use to navigate their environment remain to be fully elucidated, we have found that JNK signaling exerts fine-tune control over cell biological processes required for proper interneuron migration.

### Conclusions

Using a combination of pharmacological and genetic approaches, we found a novel requirement for JNK signaling in MGE interneuron leading process branching and nucleokinesis. Our findings are also the first to implicate the JNK signaling pathway as a key intracellular regulator of the dynamic positioning of multiple subcellular organelles involved in interneuron migration. The exact molecular mechanisms controlling JNK signaling in interneuron migration remain to be determined. Therefore, identifying the upstream activators and downstream targets of JNK signaling will provide further insight into the role of JNK signaling in cortical development and disease.

## Synthesis

Reviewing Editor: Alfonso Represa, INSERM Decisions are customarily a result of the Reviewing Editor and the peer reviewers coming together and discussing their recommendations until a consensus is reached. When revisions are invited, a fact-based synthesis statement explaining their decision and outlining what is needed to prepare a revision will be listed below.

The manuscript investigated the role of Jnk signaling for cortical interneuron migratory behaviors and provided evidence that it affects multiple modes of migratory behaviors. Using interneuron-specific, conditional genetic models of Jnk1, Jnk2, and Jnk3, pharmacological inhibition of JNK signaling, and live imaging of the interneuron, the authors show that inhibition of JNK signaling in interneurons disrupts the kinetics of leading process branching, rate and amplitude of nucleokinesis, and kinetics of the centrosome and primary cilium to the trailing process. The mechanism of how JNK signaling regulates migratory behaviors was not elucidated.

There are several important issues to be addressed:

1. In pharmacological experiments, the authors analyzed the behavior of interneurons on feeder cells of the cortex in the presence and absence of a pan-JNK inhibitor, SP600125. How can the authors exclude the possibility that the observed changes in interneuron behaviors were indirectly caused by feeder neurons, which might also be affected by SP600125?

2. In an article published earlier (Development (2020) 147, dev180646. doi:10.1242/dev.180646), results very similar to those in Figures 1 and 4 were reported. In this study, cortical slices were used but the same pharmacological agent and knockout mice were used. This needs to be clearly stated /commented in the ms.

3. The authors also genetically ablated all three JNK genes from interneurons by using mice containing the Dlx5/6-CIE transgene to conditionally remove Jnk1 from Jnk2;Jnk3 double knockout embryos (Dlx5/6-CIE;Jnk1fl/fl;Jnk2-/-;Jnk3-/-). Although they claim that the results are "similar" to those of pharmacological experiments, this is not that truthful. For example, while the migratory speed is greatly reduced by the addition of SP600125 (Figure 1D), it was virtually unaffected (Figure 4D).

4. The major effect of SP600125 is a reduction of migration speed. Other phenotypes may have resulted from delayed migration. To address this issue, the authors could compare the migratory behaviors of neurons with a similar speed between SP600125-treated and untreated cultures.

5. The methods of data sampling are not sufficiently described. For each analysis, the number of cells and the number of cultures should be clarified, and if there was a variation among cultures, it should also be described.

6. Quantification requires definitions of parameters to be quantified. Please provide the definitions of growth cone splitting, side branch, cytoplasmic swelling, and the nucleus.

7. Have authors evaluated the changes in primary cilia length in migrating MGE interneurons, in addition to their cellular localization (related to Figure 6) ?, this may help to provide a comprehensive representation of primary cilia dynamics during interneuronal migration. Similarly, inclusion of basic changes in primary cilia in conditional triple knockout (cTKO) interneurons in vivo will further strengthen the impact of this manuscript.

8. In discussion, incorporation of recent studies on cilia driven signaling mechanisms in migrating neurons (Stoufflet et al., 2019) and other neuronal migration related studies (Guo et al., 2015) would be useful.

9. Histogram data in figures must be presented as Box and Whisker, or even better scatter plot.

## Author Response

Author response:

1. In pharmacological experiments, the authors analyzed the behavior of interneurons on feeder cells of the cortex in the presence and absence of a pan-JNK inhibitor, SP600125. How can the authors exclude the possibility that the observed changes in interneuron behaviors were indirectly caused by feeder neurons, which might also be affected by SP600125?

We are unable to completely exclude the possibility that changes in interneuron migratory behavior in SP600125 experiments result from non-cell autonomous effects of JNK inhibition. It is unlikely that all influence of JNK on interneuron migration is extrinsic in origin however, since we demonstrate that genetic loss of JNK signaling exclusively from interneurons recapitulates important aspects of our pharmacological findings (Results page 14 lines 329-335). Nevertheless, phenotypic differences observed between pharmacological inhibition of JNK and genetic ablation of JNK from interneurons may arise from differences in cell intrinsic versus extrinsic requirements. These concepts are now clarified in the Discussion page 24-25 lines 575-595.

2. In an article published earlier (Development (2020) 147, dev180646. doi:10.1242/dev.180646), results very similar to those in Figures 1 and 4 were reported. In this study, cortical slices were used but the same pharmacological agent and knockout mice were used. This needs to be clearly stated /commented in the ms.

Thank you for this suggestion. We now explicitly state how the current study both corroborates and extends previously published work in the introduction page 3 lines 57-66, results page 10 lines 232-235; page 15 lines 344-346, and discussion page 24 lines 566-572 sections.

3. The authors also genetically ablated all three JNK genes from interneurons by using mice containing the Dlx5/6-CIE transgene to conditionally remove Jnk1 from Jnk2;Jnk3 double knockout embryos (Dlx5/6-CIE;Jnk1fl/fl;Jnk2-/-;Jnk3-/-). Although they claim that the results are "similar" to those of pharmacological experiments, this is not that truthful. For example, while the migratory speed is greatly reduced by the addition of SP600125 (Figure 1D), it was virtually unaffected (Figure 4D).

Results obtained from analyzing migratory properties of cTKO interneurons were indeed distinct from pharmacological inhibition, particularly with respect to migratory speed and nucleokinesis. Although branching was comparably affected between experimental manipulations, frequency of nucleokinesis, as well as overall migration speed, were not. We adjusted language throughout the manuscript to reflect these important distinctions more accurately (Abstract page 1 lines 10-12; Introduction pages 3-4 lines 71-75; Results page 16 lines 376-381; Discussion page 19 lines 452-461 and page 24-25 lines 575-595).

4. The major effect of SP600125 is a reduction of migration speed. Other phenotypes may have resulted from delayed migration. To address this issue, the authors could compare the migratory behaviors of neurons with a similar speed between SP600125-treated and untreated cultures.

In order to fully address this suggestion, we analyzed the migratory behavior of individual interneurons traveling at the same average migratory speed in control and JNK inhibited conditions. Intriguingly, we found statistically significant reductions in the distance of large nucleokinesis events and concomitant reductions in the maximum migratory speeds obtained under JNK inhibition, even when the average speed that interneurons traveled was not impaired. We also found branching anomalies in cells that traveled at similar average migratory speeds, suggesting features of nucleokinesis and branching are interrupted independent of speed following JNK inhibition. These data are contained in a completely new Extended Data figure (Fig. 1-1) and are explained in the text of the results pages 13-14 lines 306-326 and discussion page 19 lines 449-452 sections.

5. The methods of data sampling are not sufficiently described. For each analysis, the number of cells and the number of cultures should be clarified, and if there was a variation among cultures, it should also be described.

The requested information pertaining to data sampling is now more adequately described in the methods section on pages 6-8 lines 142-181; 187-191.

6. Quantification requires definitions of parameters to be quantified. Please provide the definitions of growth cone splitting, side branch, cytoplasmic swelling, and the nucleus.

Definitions of measured parameters are now explicitly stated in methods pages 7 lines 148-151; 159-164.

7. Have authors evaluated the changes in primary cilia length in migrating MGE interneurons, in addition to their cellular localization (related to Figure 6) ?, this may help to provide a comprehensive representation of primary cilia dynamics during interneuronal migration. Similarly, inclusion of basic changes in primary cilia in conditional triple knockout (cTKO) interneurons in vivo will further strengthen the impact of this manuscript.

In order to address this suggestion, we measured the length of cilia over time in cells migrating in control or JNK inhibited conditions. Interestingly, we did not find any differences in the length of cilia, irrespective of subcellular localization, between control and SP600125 treated interneurons. This finding suggests that JNK signaling is not required for the formation or maintenance of cilia, but rather for directing cilia to the correct location. These new data are now included in Fig. 7F and described in the text of the results pages 18-19 lines 436-439 and discussion page 24 lines 559-564 sections.

Although we agree that analyses of interneuron cilia in cTKO brains would strengthen the manuscript, these studies proved too challenging to include in this revised submission. The dynamic behavior of cilia makes analyses of static fixed images from in vivo brain sections difficult to quantitatively analyze and interpret. Future work will be needed to examine dynamic subcellular distribution and function of cilia in migrating cTKO interneurons.

8. In discussion, incorporation of recent studies on cilia driven signaling mechanisms in migrating neurons (Stoufflet et al., 2019) and other neuronal migration related studies (Guo et al., 2015) would be useful.

We agree and have incorporated these recent findings into our discussion section page 22 lines 516-520 and page 23 lines 549-553.

9. Histogram data in figures must be presented as Box and Whisker, or even better scatter plot.

In response to this suggestion and editorial recommendations, we have re-analyzed our data using estimation statistics as described in methods page 9 lines 196-201 and the corresponding figure legends. Histograms have been completely removed and replaced by scatter plots in each figure. For estimation statistics, data are presented in scatterplot format with confidence intervals for the mean difference between groups indicated on the right side of each graph.
