# Supplementary figures and images for: JNK Signaling Regulates Cellular Mechanics of Cortical Interneuron Migration
Source: eNeuro. 2020 Aug 20;7(4):ENEURO.0132-20.2020. doi: 10.1523/ENEURO.0132-20.2020 (PMC7642122; doi:10.1523/ENEURO.0132-20.2020)

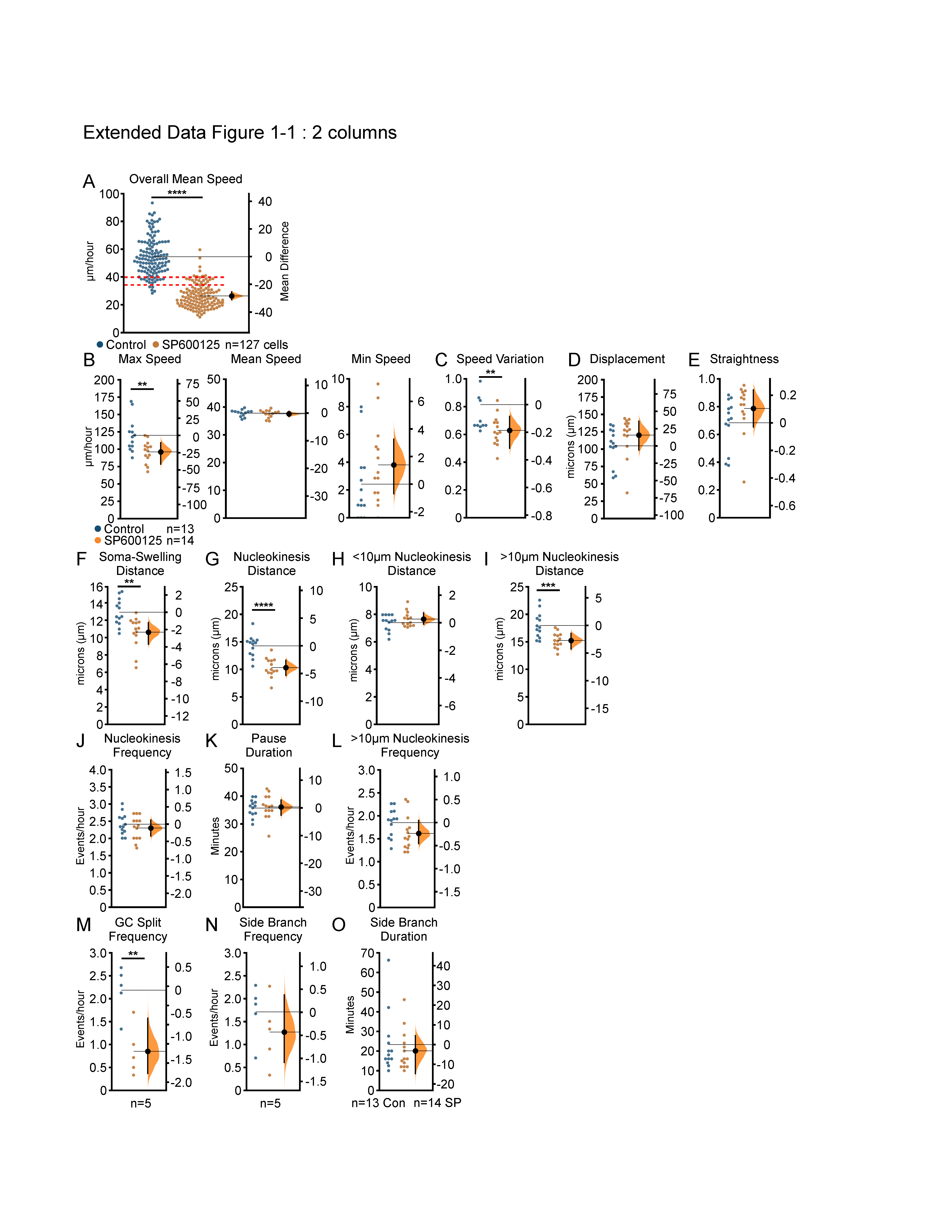

Supplement: Extended Data Figure 1-1 — JNK inhibition results in migratory deficits of cortical interneurons regardless of average migratory speed. A, Average migratory speed of individual interneurons in control and JNK inhibited conditions. Red dashed lines highlight cells migrating at the same average speed (35–40 µm/h). Data are individual data points with 5000 bootstrap sampling distribution with the mean difference between control and SP600125-treated conditions on the right y-axis. In each condition, n = 127 cells from 11 movies obtained over four experimental days. Quantification of migratory properties in interneurons migrating at 35–40 µm/h revealed significant disruptions in maximum migration speed (B), and speed variation (C), but not displacement (D), or straightness (E) during JNK inhibition. A total of n = 13 control cells and n = 14 SP600125-treated cells collected from six to seven movies over four experimental days were within the 35–40 µm/h average speed. F–L, Quantification of nucleokinesis dynamics in control and JNK-inhibition interneurons traveling at the same average migratory speed. Cortical interneurons treated with 20 µm SP600125 have significantly shorter average swelling distances (F), smaller average translocation distances (G), no change in short translocation distances (H), and a significant reduction in large translocation distances (I) compared to controls. SP600125 had no effect on average nucleokinesis frequency (J), pause duration (K), or large translocation distance frequency (L) when compared to controls. M–O, Quantification of leading process branching dynamics in control and JNK-inhibited interneurons traveling at the same average migratory speed. Interneurons treated with SP600125 have significantly reduced growth cone split frequencies (M), with no disruptions in side branch frequency (N) or duration (O). In each condition, n = 5 cells were analyzed from five movies collected over four experimental days with n = 13 control and n = 14 SP600125 side branches. Data [file enu-eN-NWR-0132-20-s03.tif]
